# Mouse obesity network reconstruction with a variational Bayes algorithm to employ aggressive false positive control

**DOI:** 10.1186/1471-2105-13-53

**Published:** 2012-04-02

**Authors:** Benjamin A Logsdon, Gabriel E Hoffman, Jason G Mezey

**Affiliations:** 1 Public Health Sciences Division, Fred Hutchinson Cancer Research Center, Seattle, Washington, USA; 2 Department of Biological Statistics and Computational Biology, Cornell University, Ithaca, New York, USA; 3 Department of Genetic Medicine, Weill Cornell Medical College, New York, New York, USA

## Abstract

**Background:**

We propose a novel variational Bayes network reconstruction algorithm to extract the most relevant disease factors from high-throughput genomic data-sets. Our algorithm is the only scalable method for regularized network recovery that employs Bayesian model averaging and that can internally estimate an appropriate level of sparsity to ensure few false positives enter the model without the need for cross-validation or a model selection criterion. We use our algorithm to characterize the effect of genetic markers and liver gene expression traits on mouse obesity related phenotypes, including weight, cholesterol, glucose, and free fatty acid levels, in an experiment previously used for discovery and validation of network connections: an F2 intercross between the C57BL/6 J and C3H/HeJ mouse strains, where apolipoprotein E is null on the background.

**Results:**

We identified eleven genes, Gch1, Zfp69, Dlgap1, Gna14, Yy1, Gabarapl1, Folr2, Fdft1, Cnr2, Slc24a3, and Ccl19, and a quantitative trait locus directly connected to weight, glucose, cholesterol, or free fatty acid levels in our network. None of these genes were identified by other network analyses of this mouse intercross data-set, but all have been previously associated with obesity or related pathologies in independent studies. In addition, through both simulations and data analysis we demonstrate that our algorithm achieves superior performance in terms of power and type I error control than other network recovery algorithms that use the lasso and have bounds on type I error control.

**Conclusions:**

Our final network contains 118 previously associated and novel genes affecting weight, cholesterol, glucose, and free fatty acid levels that are excellent obesity risk candidates.

## Background

Network analysis algorithms have been applied to genome-wide polymorphism and gene activity data to identify molecular pathways that mediate risk for complex diseases [1-5]. Such analyses have led to the discovery of novel network connections that have been subsequently validated by experiment. For example, Yang et al. [6] validated three novel genes involved in obesity and obesity related phenotypes in an F_2 _mouse cross, based on predictions made from network analysis of genome-wide data. While there have been a few successful validations of this type [6,7], it has been noted that the false discovery rates of most network analysis techniques are still unacceptably high, given the significant time, financial, and resource investment required for such validation experiments [3]. This is a problem for all current statistical network modeling approaches, whether focused on ensemble behavior of groups of genes [1,4,8-10], specific conditional network interactions among genes [11-14], or directed networks [15-20]. For both broad pattern and specific network modeling methods, there can be high false discovery rates due to random noise and systematic error among samples, unless these are correctly accounted for in the experimental design or underlying statistical modeling framework [21].

We propose a novel algorithm that is able to directly control both systematic error and over-fitting sources of high false discovery rates in network reconstruction. The method balances the need for a network modeling methodology with an aggressively controlled false discovery rate, that is capable of representing rich statistical dependencies. To control false discovery rate, the method uses a regularized regression framework for undirected network inference [12,13,22] by employing a spike-and-slab prior on the regression coefficients [23] along with a probabilistic consistency bound on the model size [24]. The spike-and-slab has been conjectured to approach optimal estimation for sparse models [24,25], and does not suffer from the irrepresentability condition that is a property of many popular penalties, such as the lasso [26], where the wrong model can be returned even asymptotically [27]. By using a Bayesian framework, the mixture proportions of the prior are estimated directly from the data, negating the need for penalty selection by cross-validation or information theoretic model selection, as with other penalized approaches [22,28]. For scaling purposes, the full algorithm makes use of a variational Bayes approximation to allow Bayesian model averaging when considering very large sets of putative network features (i.e. tens of thousands to millions) [29]. This approach results in the algorithm returning a sparse network model in which all connections have strong statistical support, instead of a model where only the top few are expected to have a low false discovery rate. To control possible sources of systematic, or confounding error, our method also incorporates the top eigenvectors from a principal component analysis as unpenalized coefficients, an error controlling approach that has been successful in related applications [21,30].

We demonstrate the strength of our methodology by analyzing genotype, gene expression, and downstream phenotype data from the F2 intercross generated from mouse strains C57BL/6 J and C3H/HeJ with apolipoprotein E as null on the background (BxH.ApoE^-/-^) [9,31] to identify network connections among genes and obesity related phenotypes. The genome-wide data from this cross have been used to generate large-scale network predictions of genetic interactions affecting metabolic syndrome associated phenotypes [1,6,9,31] and have been used as a foundation for experimental validation of predicted network connections between genes and obesity [6]. On a practical level, this experiment has a sufficient sample size (298 F_2 _progeny) to justify the use of a rich statistical model. We demonstrate that our algorithm performs better than the popular lasso [32] and the adaptive lasso [33] penalized regression approaches, by demonstrating that neither approach can return a sparse model, where all variables are strongly supported, when using approaches to bound Type I errors or a standard cross-validation choice of penalty parameter. The improved control of false positives with our variational Bayes algorithm is a direct consequence of the forced sparsity of the solution which is induced by the specification of a probabilistic bound on the model size within the algorithm as a function of the sample size and number of variables.

### Theory - undirected network models

A probabilistic undirected network model is defined as an undirected graph with an associated probability measure [34]. An undirected graph G is specified as a pair $G=\left\{V,E\right\}$,with V a set of vertices (i.e. gene expression products, single nucleotide polymorphisms, or downstream phenotypes), and *ℰ *a set of unordered pairs of vertices, specifying undirected edges between vertices [34]. We focus on a type of Gaussian graphical model (GGM) defined with respect to a conditional multivariate normal distribution, where we model the distribution of expression traits, conditional on the genotypic states (see Methods for parametric details of a conditional GGM). This particular type of model only captures linear interactions between variables (we do not consider nonlinear or epistatic interactions in this paper). The goal of any network discovery algorithm is to determine the relevant set of edges for each vertex in the graph (i.e. the neighborhood of other vertices connected to each vertex), based on the observed genetic polymorphism, expression, and downstream phenotype data. Most practical genomics applications have far more variables (i.e. vertices) than samples, and therefore require a carefully constrained solution to the neighborhood selection problem, to prevent over-fitting and high false discovery rates [3,12]. We therefore focus our attention on the regularized solutions to this problem [12-14,22], where the identified neighborhood size can be restricted based on the choice of penalization, and where the neighborhood selection problem is solved through penalized regression of each phenotype on all other phenotypes and genotypes [12,13,22].

### Bayesian spike-and-slab prior sparse feature selection and accounting for systematic error

In our algorithm, we treat the neighborhood selection problem for any given phenotype *y *as a regularized regression problem. More specifically, this neighborhood selection problem involves identifying a subset of expression products or genotypes with non-zero regression coefficients in a multiple regression equation *y_i _*= *μ *+ ∑*_j _x_ij _β_j _*+ *e_i_*, where a penalty is defined over the regression coefficients (*β*_1_,...,*β*_*p *+ *m*_) for the *i^th ^*sample with *j *= 1,...,*p *+ *m *possible expression phenotypes and genotypes. We use a mixture spike-and-slab prior as our penalty, $\beta_{j}\sim p_{\beta =0}\text{I}\left[\beta =0\right]+p_{\beta \neq 0}N\left(0,\sigma_{\beta }^{2}\right)$, with the spike (*p*_β = 0_I[*β *= 0]) being related to an *l*_0 _type penalty which drives the sparse feature selection and the slab $\left(p_{\beta \neq 0}N\left(0,\sigma_{\beta }^{2}\right)\right)$ being related to a ridge or $l_{2}^{2}$ type penalty which effectively smooths the identified sparse model. The combination of the two different types of penalization is similar in principle to the elastic net penalty [35], which incorporates a combination of *l*_1 _and $l_{2}^{2}$ penalties. There is both theoretical [27] and empirical evidence [29] that the spike-and-slab prior is more effective than other penalties such as the lasso [12,13] at generating sparse solutions with low false discovery rates, for ultra-high-dimensional problems when p ≫ n, where *p *is the number of variables, and *n *is the sample size.

We employ a fully Bayesian framework to handle the spike-and-slab prior, where the mixture proportions are estimated directly from the data. The algorithm finds an appropriate level of sparsity supported by the data via the probabilistic bound on model size without relying on cross-validation type approaches or information theoretic model selection criterion. Specifically, this is done by constraining the model dimension such that the number of selected features (*s*) for any given problem are on the order $s\left(n\right)=O\left(\sqrt{n}\right)$. This is done by truncating the distribution of *p*_*β*≠0 _such that $p_{\beta \neq 0}\leq \sqrt{n}/\left(m+p-1\right)$ for *p *gene expression or downstream phenotypes and *m *genotypes. Given mild regularity conditions it has been shown in the context of linear regression that consistency can be established for both $s\left(n\right)=o\left(\sqrt{n}\right)$[36] and under further mild assumptions *s*(*n*) = *o*(*n*/log(*n*)) [37]. Note that the latter bound is a much weaker constraint on the model size as a function of the number of observations than the former bound. In addition, Zhang et al. [25] show that the $s\left(n\right)=O\left(\sqrt{n}\right)$ choice of model size will asymptotically lead to minimum prediction error at a rate $O\left(n^{-1/2}\right)$. This justifies our choice of the strength of the penalization to be sufficiently conservative in terms of ensuring few irrelevant features enter the model when p ≫ n, especially for data with at least hundreds of observations, because of the optimal rate. This bound is also consistent with the results from the simulations within this paper, as well as results from previous applications of this bound [24,25,29]. The Bayesian framework also allows the algorithm to take advantage of the multiple modality of the posterior with Bayesian model averaging, a particularly valuable approach when any well-supported sparse solution is expected to capture only a portion of the true network connections.

Another feature of our algorithm is that we also simultaneously correct for systematic error, or other large scale confounding factors among samples, based upon the expression data, by including the top twenty eigenvectors from a principal component analysis as unpenalized fixed effects in our model selection procedure. Therefore, the previous multiple regression equation becomes *y_i _*= *μ *+ ∑*_j_ x_ij _β_j _*+ ∑*_k _t_ik _α_k _*+ *e_i_*, with *t*_1_,...,*t*_20 _being the top 20 across sample eigenvectors obtained from a standard principal component analysis of the joint gene expression data. The motivation for this is analogous to the use of eigenvectors from principal component analysis to correct for confounding population structure in genetic association analyses [21,30], which aims to remove any potentially confounding effects from the inference of the neighborhood of any given phenotype.

### Variational Bayes approximate inference

**Figure 1:**
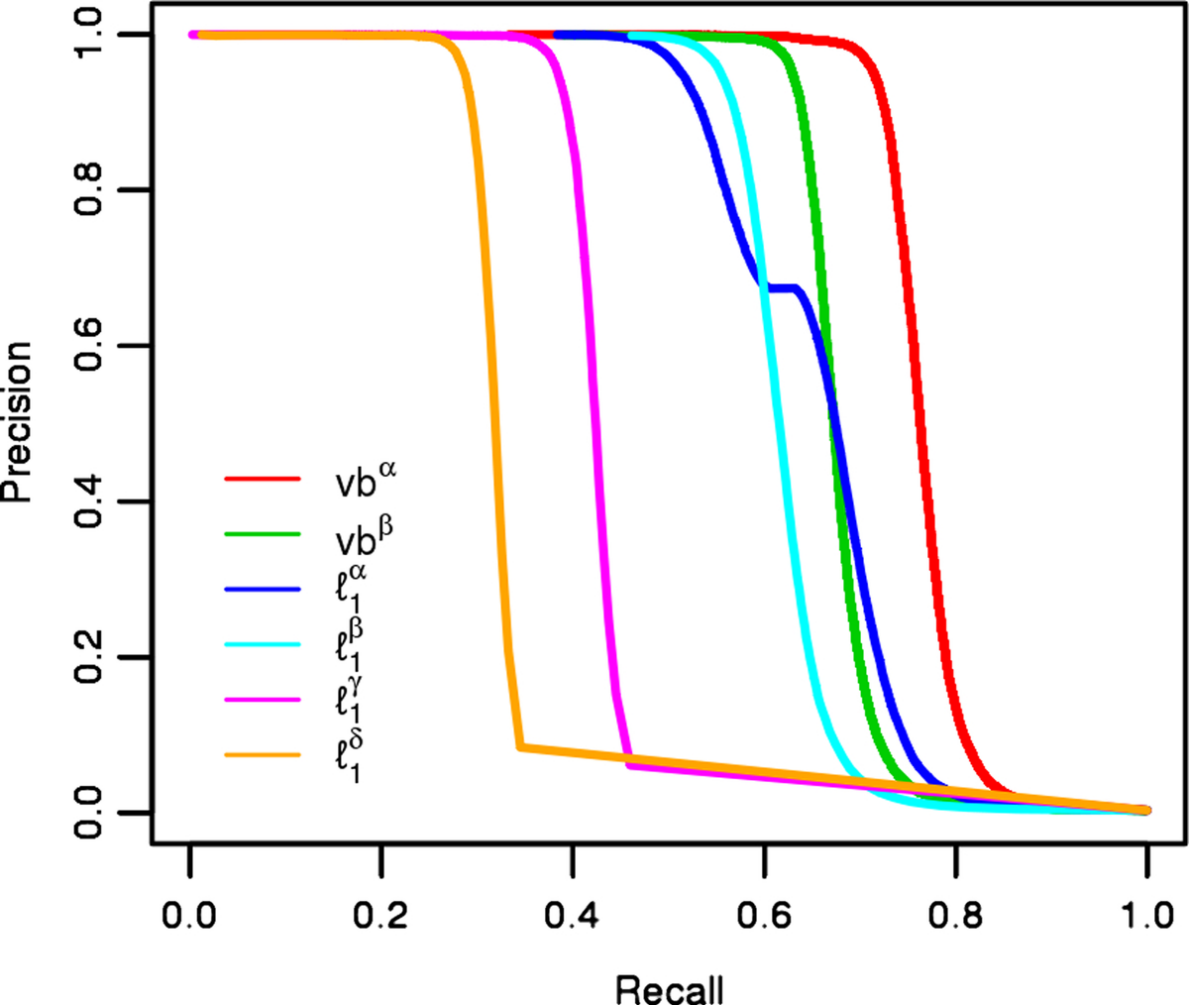
**Precision-recall curves for simulated networks**. Precision-recall curves for different strategies for setting the significance threshold for the variational Bayes method as a function of the posterior probability and the randomized lasso with stability selection as a function of the empirical recovery probabilities for stability selection. Twenty replicate networks with 1000 variables, 300 observations, and an average of 1.47 edges per node were simulated (see Methods for further details). The network reconstruction methods compared are as follows, vb*^α^*: variational Bayes method with posterior probabilities averaged in both directions of regression, vb*^β^*: variational Bayes method with posterior probabilities not averaged, $\ell_{1}^{\alpha }$: randomized lasso with stability selection with the number of false positives bounded below 1 and recovery probabilities averaged in both directions of regression, $\ell_{1}^{\beta }$: same as $\ell_{1}^{\alpha }$, except without averaging, $\ell_{1}^{\gamma }$: randomized lasso with stability selection with the penalty parameter chosen such that the number of false positives are bounded below 1000 and recovery probabilities averaged in both directions of regression, and $\ell_{1}^{\delta }$: same as $\ell_{1}^{\gamma }$, except without averaging.

**Figure 2:**
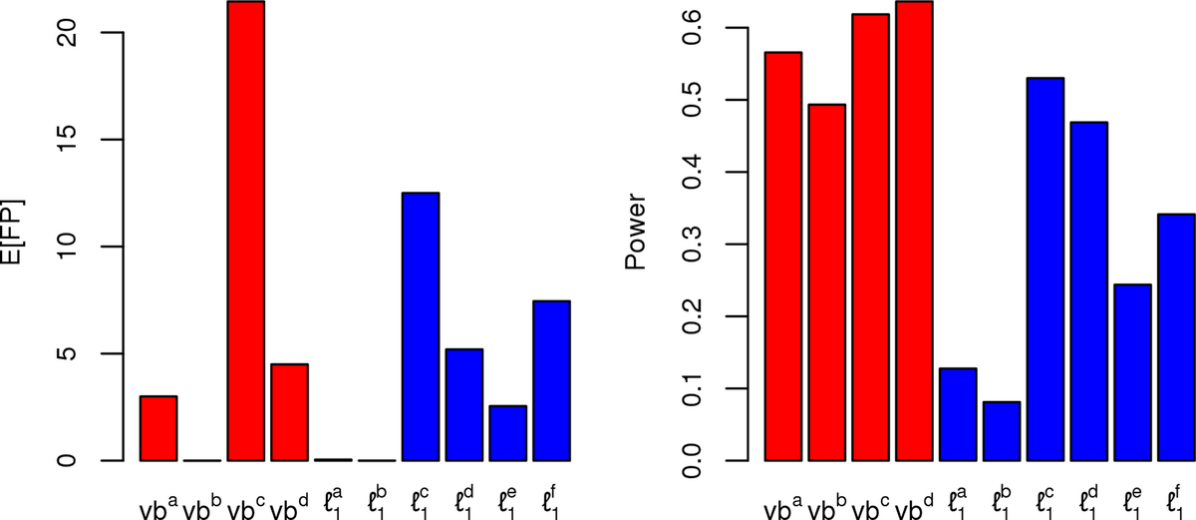
**Type I errors and power for simulated networks**. The left panel shows the average number of false positives per replicate simulation (out of twenty simulated networks as in Figure 1) for different strategies for setting the significance threshold for both the variational Bayes method and the lasso methods. The right panel shows the corresponding power for all of the methods. The methods compared are as follows, vb^a ^and vb^b^: variational Bayes methods without and with averaging in both directions of regression, and a posterior probability threshold of ${\hat{p}}_{j}>0.99$, vb^c ^and vb^d^: variational Bayes methods without and with averaging, and a posterior probability threshold of ${\hat{p}}_{j}>0.5$, $\ell_{1}^{\text{a}}$ and $\ell_{1}^{\text{b}}$: randomized lasso with stability selection with the number of false positives bounded below 1 for the entire network, without and with averaging, $\ell_{1}^{\text{c}}$ and $\ell_{1}^{\text{d}}$: randomized lasso with stability selection with the number of false positives bounded below 1000 for the entire network, without and with averaging, and finally $\ell_{1}^{\text{e}}$ and $\ell_{1}^{\text{f}}$: regular lasso with the number of false positive bounded below 1 and 1000 for the entire network.

As in Logsdon et al. [29], we use a variational Bayes approximate inference approach to solve the high-dimensional feature selection problem. For the feature selection problem, the variational Bayes approximation is a good tradeoff between speed, since it is much faster than alternative exact inference approaches, and quality of the identified solutions, where empirical evidence shows that it performs well for underlying sparse models [29]. The variational Bayes approximation consists of minimizing the Kullback-Leibler divergence between an approximate factorized posterior distribution $q_{\beta_{1}}\left(\beta_{1}\right)\cdots q_{\beta_{m}}\left(\beta_{m}\right)q_{p_{\beta \neq 0}}\left(p_{\beta \neq 0}\right)q_{\sigma_{e}}\left(\sigma_{e}\right)q_{\sigma_{\beta }}\left(\sigma_{\beta }\right)$ and the full posterior distribution *p*(*β*_1_,...,*β_m_*, *p*_*β *≠ 0_, *σ_e_*, *σ_β_*), using iterative expectation-type steps as in an Expectation-Maximization algorithm [38,39]. The relevant statistic for the *j^th ^*expression product or genotype produced by the algorithm for the problem of feature selection is the posterior probability of inclusion in the model denoted a ${\hat{p}}_{j}$ from thereon. This ${\hat{p}}_{j}$ parameter comes from the approximate posterior inference of the mixture parameters in the spike-and-slab prior. A detailed description of this statistic in terms of the other model parameters is given in the Additional file 1, Equations 2, 4, and 17. In our approach we perform a two-step reconstruction of the joint genotype, expression, and downstream phenotype network, where we first perform neighborhood selection for each downstream phenotype individually on all expression traits and genotypes. Then, in the second step, we perform neighborhood selection for each expression trait on all other expression traits and genotypes. The procedure is split into two steps because of the primary interest in the neighborhoods of the downstream phenotypes, followed by interest in the expression Quantitative Trait Loci (eQTL) networks associated with the neighborhoods of the downstream phenotypes. To resolve discrepancies in neighborhoods identified in two directions of regression, we average the ${\hat{p}}_{j}$ scores across both directions of regression at a cutoff of ${\hat{p}}_{j}>0.99$. This approach is supported by the significant improvement in results obtained from simulations (see Figures 1, 2). We then combine the neighborhoods of the first and second step of the algorithm through a simple union operation.

## Results

### Simulation results

We performed a simulation study to compare our approach to other comparable methods that use the lasso with mechanisms for bounding the number of type I errors, as well as to lasso methods using a standard cross-validation approach, shrinkage estimation, partial least squares estimation, and ridge estimation methods. For the bounded type I error lasso methodologies, this included the randomized lasso with stability selection [40] and the regular lasso with the penalization chosen to bound the number of type I errors as in Meinshausen and Bühlmann [12]. We simulated twenty networks with a random underlying topology, *p *= 1000 variables, *n *= 300 observations, and on average 1.47 edges per variable (further details of the simulation are presented in the Methods). In Figure 1 we show the precision-recall curves for two different strategies for defining the posterior probability of edge inclusion for the variational Bayes methodology: vb*^α ^*where the posterior probabilities are averaged in both directions of regression, and vb*^β^*, where the posterior probabilities are not averaged in both directions of regression. We also show four different strategies for defining the empirical selection probabilities defined by the randomized lasso with stability selection: $\ell_{1}^{\text{a}}$ where the penalization parameter is chosen as in Meinshausen and Bühlmann [40] to bound the number of false positives to be less than one, and the empirical selection probabilities are averaged in both directions, $\ell_{1}^{\beta }$ where the penalty parameter is chosen similarly, but the empirical selection probabilities are not averaged, $\ell_{1}^{\gamma }$ where the penalization parameter is chosen as in Meinshausen and Bühlmann [40] to bound the number of false positives to be less than 1000, and finally $\ell_{1}^{\delta }$, where the penalty parameter is chosen similarly, but the empirical selection probabilities are not averaged. All curves are generated as a function of the threshold for declaring significance based on the associated probability statistics. We see that the variational Bayes approach significantly outperforms the randomized lasso with stability selection in terms of both power and type I error control for the averaged and non-averaged (vb*^α^*, vb*^β^*) posterior probability statistics across most thresholds for declaring significance.

In Figure 2, we illustrate the performance in terms of the average number of false positives observed in the entire network per replicate network (the left panel), and the overall power (the right panel) for specific thresholds of the variational Bayes and lasso approaches. Specifically, we investigate the variational Bayes approach for the conservative posterior probability thresholds of ${\hat{p}}_{j}>0.99$, not averaged (vb^a^), and averaged (vb^b^), as well as for the more liberal ${\hat{p}}_{j}>0.5$, not averaged (vb^c^), and averaged (vb^d^) as described above. We also show the randomized lasso with stability selection for the more conservative strategy where the number of false positives is bounded to be less than 1, not averaged ($\ell_{1}^{\text{a}}$), and averaged ($\ell_{1}^{\text{b}}$), as well as for the more liberal strategy of the number of false positives bounded to be less than 1000, not averaged ($\ell_{1}^{\text{c}}$), and averaged ($\ell_{1}^{\text{d}}$). Finally, we show the method of choosing the penalization of the regular lasso to bound the number of false positives to be less than 1 [12] ($\ell_{1}^{\text{e}}$), and less than 1000 ($\ell_{1}^{\text{f}}$). Across all of these results, we see that the more conservative variational Bayes approaches (vb^a ^and vb^b^) outperforms the conservative lasso approaches ($\ell_{1}^{\text{a}}$, $\ell_{1}^{\text{b}}$, and $\ell_{1}^{\text{e}}$), as well as most of the more liberal lasso approaches (except with vb^b ^and $\ell_{1}^{\text{c}}$), while at the same time recovering far fewer false positives. At the more liberal threshold of ${\hat{p}}_{j}>0.5$, the performance of the variational Bayes algorithm is further improved, especially for the averaged solution, vb^d^, which has a comparable number of false positives to any of the liberal lasso solutions, but has much greater power. The lasso methods had the most comparable performance to our algorithm based on additional simulated data considering five competing methods: lasso, adaptive lasso, shrinkage estimation [14], partial least squares estimator [41], and a ridge estimator [22] (Figures 1, 2, Additional file 1: Figure S4, Additional file 1: Figure S5, Additional file 1: figure S6, Additional file 1: figure S7) (see the Additional file 1 for a detailed description of additional simulations and network reconstruction methods). Therefore we only compared the lasso approaches to our algorithm when analyzing the experimental data.

### Mouse downstream phenotype neighborhood identification

For the data analysis, we analyzed the F_2 _progeny of a cross between the C57BL/6 J (B6) and C3H/HeJ (C3H) strains on an apolipoprotein E null (ApoE -/-) background (BXH.ApoE^-/-^), as presented in Ghazalpour et al. and Wang et al. [9,31]. We focused on the gene expression data that was collected in the liver of the mice where expression was assayed on 23,574 custom probes [9]. In addition, there were 22 downstream phenotypes that were assayed, including weight, cholesterol, glucose, free fatty acid, among other metabolic phenotypes, as well as 1,347 genetic markers [9]. A total of 298 individuals were retained after filtering down to those for which both expression and genetic markers were collected. Previous authors have shown the antagonistic sex effects within this data [31], i.e. the effect of a risk locus is opposite between males and females. To address the sex specific effects, as well as other possibly confounding factors, we included both the sex and the 20 first eigenvectors from a principal component analysis computed across samples for expression phenotypes as unpenalized fixed effects in our linear model for all methods that we compared.

We first ran our variational algorithm on each of the 22 obesity related downstream phenotypes individually, where we performed sparse feature selection on all gene products and genetic markers. Our variational algorithm produced a sparse set of expression and genetic markers for each downstream phenotype, with the phenotypes with more than seven expression or genotype features identified shown in Additional file 1: Table S1. We ran the randomized lasso with stability selection to bound the number of false positives to be less than 1 [40], and the regular lasso with the choice of penalty to bound the false positives to be less than 1 [12], and found that no expression traits or genotypes were identified as having non-zero effects by either approach. We also ran the lasso and adaptive lasso with ten-fold cross-validation for the same set of downstream phenotypes, as shown in Additional file 1: Table S1. The number of identified genetic interactions of each downstream phenotype was on average much larger for the lasso and the adaptive lasso with ten-fold cross-validation. The variational algorithm identifies additional features, with only 55% overlap with the lasso, and 46% overlap with the adaptive lasso for the seven phenotypes shown in Additional file 1: Table S1.

**Figure 3:**
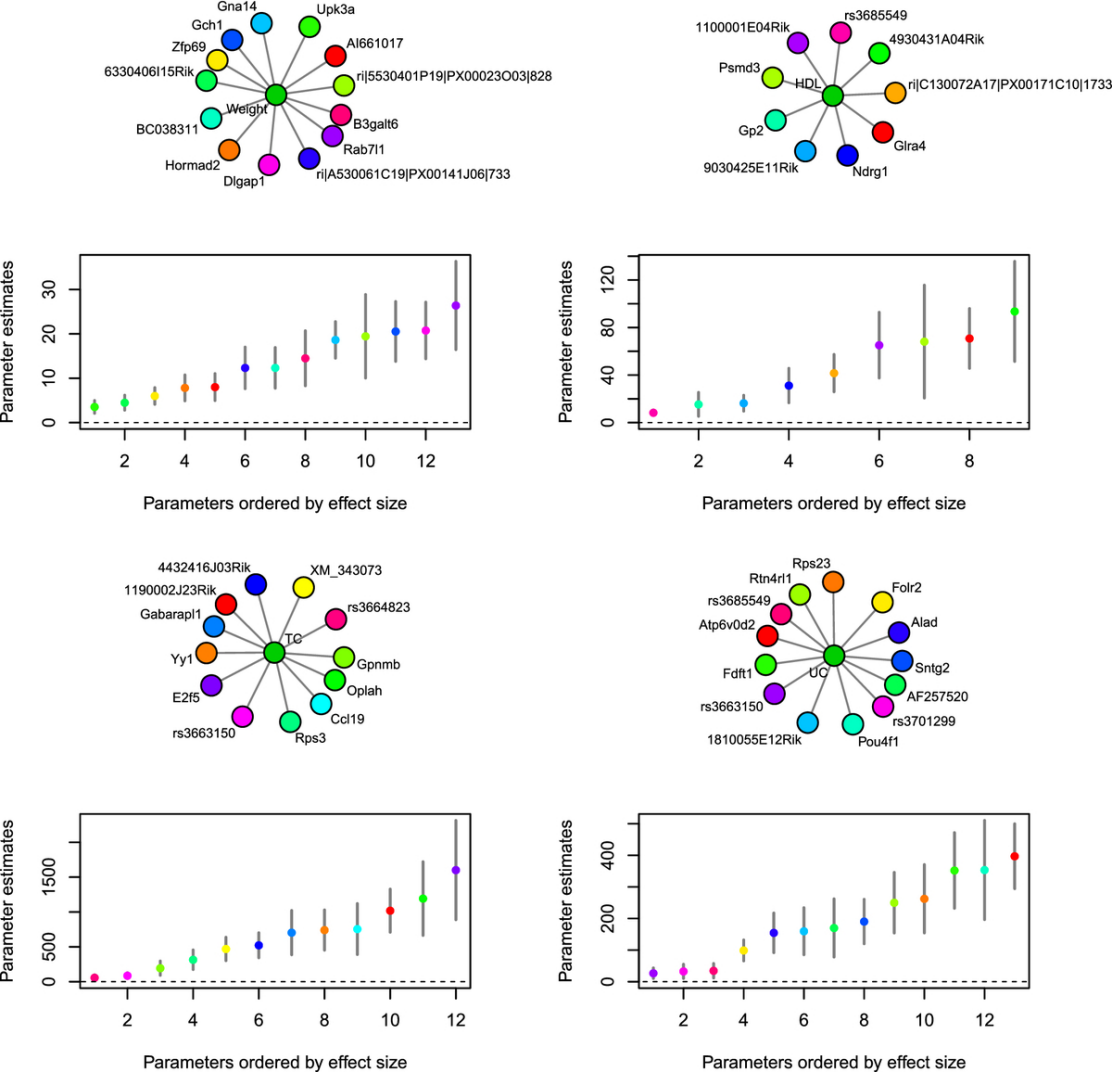
**Local networks for obesity phenotypes**. Network graph models of the genetic markers and gene expression traits identified by the variational Bayes algorithm as being associated with the downstream phenotypes: weight, high-density lipoprotein cholesterol levels (HDL), total cholesterol levels (TC), unesterified cholesterol (UC) levels. The plots below present the ordered estimates and 0.95 confidence intervals for the parameters from an independently fit, unpenalized multiple regression model of the downstream phenotypes on the genetic markers and gene expression trait in each of the identified models.

**Figure 4:**
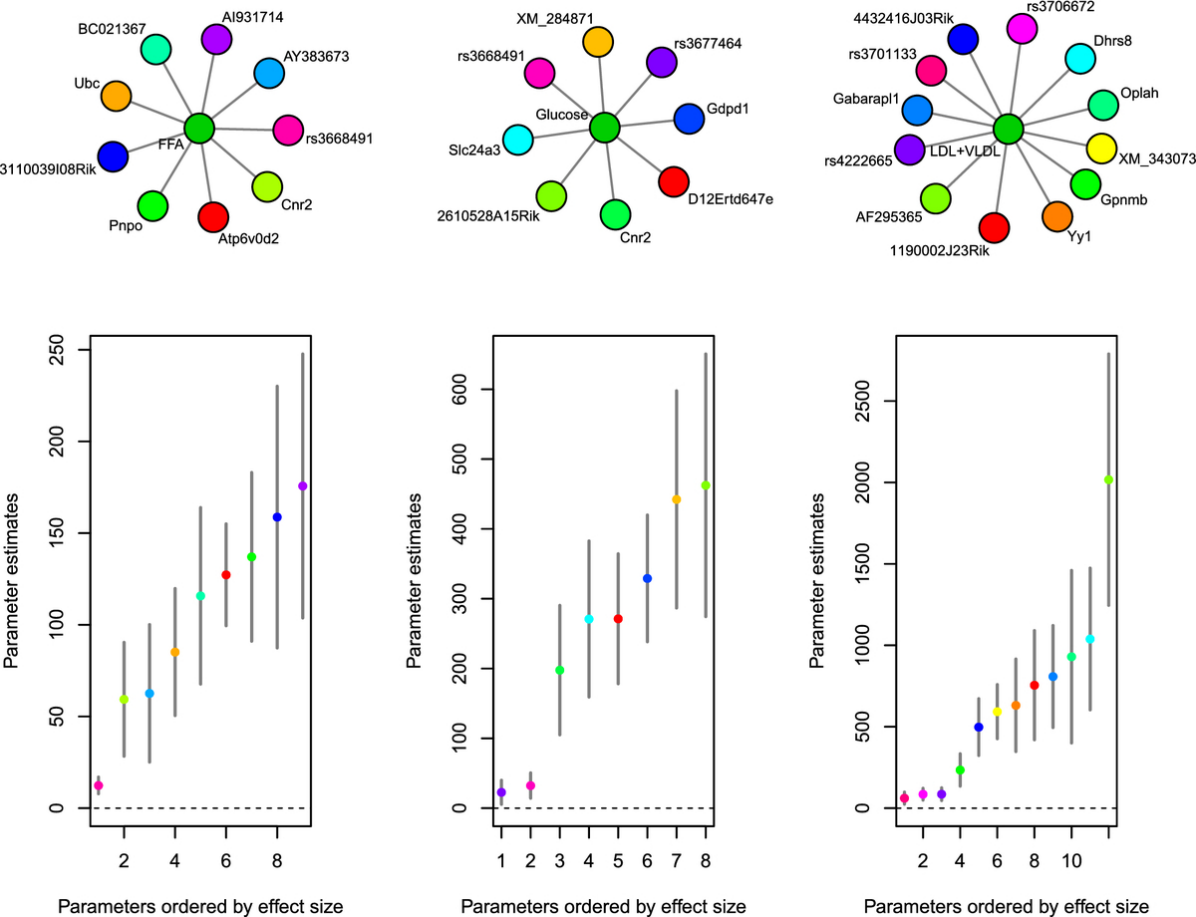
**Local networks for obesity phenotypes**. Network graph models and confidence interval plots as described in Figure 3 for free fatty acid (FFA) levels, glucose levels, and low-density lipoprotein + very low-density lipoprotein (LDL + VLDL) levels.

To assess the statistical confidence of our initial mouse obesity analysis, we determined the confidence intervals for each of the downstream phenotype network connections recovered with each feature selection method, in an independent, non-penalized linear multiple regression model. Both the lasso and the adaptive lasso contained many features that were not statistically significant at the *P *< 0.05 significance level, indicating that the use of cross-validation as a method to control the sparsity of the model for the lasso or adaptive lasso allows an unacceptable number of false positives to be included in the model. We depict the network model and confidence intervals for the network connections identified by the lasso and adaptive lasso for weight in Additional file 1: Figure S1 and Additional file 1: Figure S2. For all phenotypes, the features identified as having a downstream network connection by the variational algorithm were all significant (the models and confidence intervals are depicted in Figures 3 and 4). We also recapitulated a similar result in additional simulations where we demonstrate that at the ${\hat{p}}_{j}>0.99$ cutoff, the variational Bayes method returns fewer false positives and tighter confidence intervals on all predicted network connections as opposed to the lasso and adaptive lasso (Additional file 1: Figure S3). Given the increased performance in terms of learning a statistically robust model and the appropriate sparsity of that model, we proceeded with only the variational Bayes algorithm for the expanded network analysis.

### Expanded undirected network reconstruction

**Figure 5:**
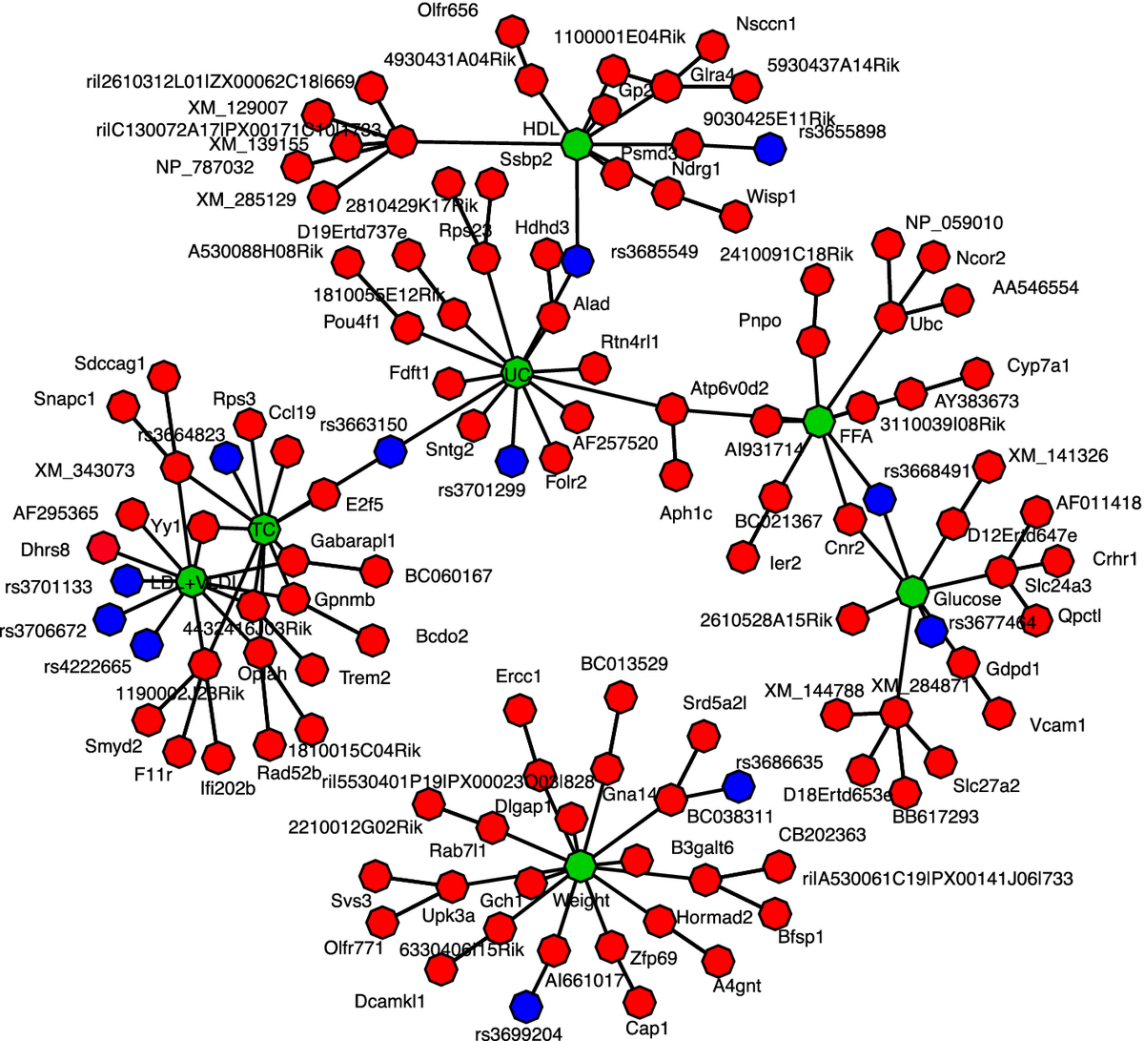
**Integrated obesity network**. An undirected obesity and metabolic network reconstructed from the mouse F_2 _intercross data using the variational Bayes algorithm. The network depicts genetic markers (blue), gene expression traits (red), and the downstream phenotypes (green) weight, high-density lipoprotein cholesterol levels (HDL), total cholesterol levels (TC), unesterified cholesterol (UC) levels, free fatty Acid (FFA) levels, glucose levels, and low-density lipoprotein + very low-density lipoprotein (LDL + VLDL) levels.

In the second step of our analysis, we used our variational Bayes algorithm to generate an undirected network among genotypes, expressed genes, and downstream phenotypes, by solving the neighborhood selection problem for each gene expression product individually, against all other genes and genetic markers. We resolved the neighborhoods of the networks very conservatively, by averaging the ${\hat{p}}_{j}$ scores in both directions of regression for the expression phenotypes, and only declaring an interaction between genes present in the model if the averaged ${\hat{p}}_{j}$ scores were greater than 0.99. To determine the most relevant aspects of this sparse network with respect to weight and other related phenotypes, we combined the neighborhoods produced for each of the downstream phenotypes from the first phase of the analysis and the second phase expression undirected network, to depict the local sub-networks associated with each downstream phenotype, for weight, total cholesterol, high density lipoprotein (HDL) cholesterol, unesterified cholesterol (UC), free fatty acids (FFA), glucose levels, and low density lipoprotein + very low density lipoprotein (LDL + VLDL) levels (Figure 5). Table 1 summarizes identified genes which have been previously implicated in obesity, or related diseases and pathologies (Additional file 1: Table S2 is a version of this table with references available in the Additional file 1).

**Table 1:** Obesity related interactions.

Gene/SNP	Disease	Organism(s)
Zfp69	Candidate gene for diabetes associated with obesity	Mouse and Human

Gna14	Association study of hypertension	Human

F11r	Induces hypertension in the brain stem	Rat

Gabarapl1	Regulator of insulin dependent hepatic autophagy	Mouse

Wisp1	Association study of hypertension	Human

Fdft1	Squalene (cholesterol) biosynthesis gene	Mouse and Human

Ier2	Induced gene in insulin signalling pathways	Rat

Slc24a3	Down regulated in diet sensitive obesity	Human

Crhr1	Candidate obesity gene possibly affecting feeding behavior	Mouse and Human

Qpctl	Association study identified candidate obesity gene	Human

Vcam-1	Atherosclerotic plaque associated gene	Human

Gch1	Identified in linkage studies of maximal sedentary oxygen uptake	Human

Dlgap1	Type-2 diabetes associated gene	Human

Yy1	Type-1 diabetes associated gene	Rat

Ccl19	Adipocyte inflammation	Human

Cnr2	Obesity associated adipocyte inflammation	Mouse

Atp10a/rs3664823	Obesity associated gene	Mouse

Folr2	Up-regulated in obesity associated adipose tissue	Human

Interactions identified by the variational method with previous evidence of being associated with obesity, or obesity related phenotypes

The network recovered by our algorithm is enriched for interactions that have been previously associated with these phenotypes: a total of 18 out of 118 recovered. While this may appear modest, it still suggests that this list of 118 variables is enriched for good candidate genes for follow up studies. From the first step of the analysis we find eleven genes (Gch1, Zfp69, Dlgap1, Gna14, Yy1, Gabarapl1, Folr2, Fdft1, Cnr2, Slc24a3, and Ccl19), as well as a single nucleotide polymorphism (rs3664823) that are directly linked to the downstream metabolic phenotypes and have independent evidence of being associated with obesity or obesity related pathologies (along with 52 novel genetic variables). We further identify six genes that feed into the genes that directly interact with the metabolic phenotypes (Ier2, H11r, Wisp1, Crhr1, Qpctl, Vcam-1), as well as an additional 54 novel interactions. These other novel interactions included Dcamkl1, Ercc1, and Cyp7a1, implicating a possible connection with weight, intestinal stem cell lineage [42], and DNA damage repair [43], along with a connection between the levels of free fatty acids and bile acid production [44].

## Discussion

Our variational Bayes algorithm is designed as a scalable and robust method for recovering a sparse network from the analysis of genome-wide data where only statistically relevant features are returned. This makes it particularly well suited to analyses aimed towards experimental validation of predicted biological interactions, for which the burden of false positives is costly [3]. While our algorithm does not provide a large-scale picture of network topology that is the goal of the majority of network analysis methodologies [11-19], it nevertheless provides a short list of very statistically significant features, an outcome advantageous to the experimentalist interested in following up on the highest quality predictions. In addition, we analyzed the entire network of 24,921 variables in 48 hours on a single machine with dual quad-core Intel Xeon processors (fitting a model with 3.08 × 10^8 ^possible linear interactions). Recent work has shown that the variational Bayes approach for the spike-and-slab regression model is orders of magnitude faster than the corresponding Markov chain Monte Carlo approach [45], indicating that we are able to solve high-dimensional problems much faster than the corresponding exact inference approach.

Our algorithm focuses on the reconstruction of undirected networks because they are more amenable to highly scalable, sparse feature selection methods [22]. Only a few of the undirected graphical algorithms that have been proposed, such as the lasso, that can simultaneously return sparse network models and analyze the entirety of genome-wide variables without requiring a step-wise procedure (e.g. the PC-algorithm [17]). We demonstrated through simulations that the lasso with type I error control [12,40] does not perform as well as the variational spike-and-slab approach that we propose, as shown in Figures 1, 2. In addition, when we applied the lasso with type I error control to the mouse data, we saw that the performance was even worse than in the simulations, with both stability selection and a choice of penalization to bound the type I error being severely under-powered (returning the null model for all analyses). This was not entirely surprising, given similar results in the context of genome-wide association studies for the lasso with stability selection [46], where high-dimensional data with p ≫ n and significant correlations among variables caused stability selection to perform very poorly in terms of power (likely because of the difficulty in deciding which variable within a given correlated block of variables should be included in the model). However, the lasso and adaptive lasso with cross-validation were able to identify non-null models for the downstream phenotypes, though these methods may not necessarily produce solutions that are highly enriched for true positives. We find that while the lasso and adaptive lasso with cross-validation can produce some quality predictions of biological interactions, they also include a majority of statistically less supported results compared to our variational Bayes algorithm, which is able to estimate both a strongly statistically supported model, and the degree of sparsity of the model.

It should be noted that despite the similarities between the model we propose in this paper, and previous work for variational Bayes algorithms with spike-and-slab priors [29,45], there remains some important distinctions. First, as opposed to Logsdon et al. [29], we only consider a single non-zero component for the slab in the spike-and-slab prior, instead of two truncated normal distributions, therefore reducing the dimensionality of the parameter space. Second, we incorporate a Bayesian model averaging procedure that increases the stability of the solutions identified across the space of possible models. By down-weighting variables that are inconsistent between models with similar fits to the data, Bayesian model averaging may also address some of the concerns of Carbonetto and Stephens [45] with regards to the variational Bayes approach incorrectly identifying false positives that are correlated with true positives. Third, we simplify the estimation of the effect of unpenalized covariates by treating them as non-random parameters whose effects are estimated through the maximization of the lower bound. Finally, because we are reconstructing networks among multiple phenotypes, we show we can improve the performance of our approach by averaging the posterior probability statistics in both directions of regression (Figures 1, 2).

In addition, 5 of the 11 genes and single nucleotide polymorphism with independent evidence of possible metabolic functionality in the downstream phenotype analysis were uniquely identified by the variational spike-and-slab method but not found by either the lasso or adaptive lasso with cross-validation (Gabarapl1, Dlgap1, Folr2, Cnr2, and rs3664823). This indicates that even when the lasso is tuned to be more liberal, as with cross-validation, the variational spike-and-slab methodology can identify additional high confidence results within a particular data-set. There is evidence that this is the case because of both the non-convex nature of the spike-and-slab penalty, which does not over-penalize true effects as severely as the lasso [26], and because of the additional regularization associated with Bayesian model averaging. Bayesian model averaging effectively regularizes over the ensemble of identified solutions to only include effects with strong evidence across model space.

We identified 18 genetic variables that have been previously linked to obesity or obesity related phenotypes using our variational method with a strict control of false discovery rate. These variables include genes related to cholesterol biosynthesis such as the gene farnesyl diphosphate farnesyl transferase 1 (Fdft1). This is a known squalene (i.e. cholesterol) synthesis gene where high levels of this gene are known to be associated with visceral obesity [47]. It has also been shown to be up-regulated in mice on a high fat diet [48] and is directly linked to the unesterified cholesterol levels. We also found genes related to neurological regulation of appetite including Cnr2 and Crhr1 [49,50], and variables involved in insulin signaling pathways including the gene immediate early response 2 (Ier2) also known as Pip92, which is known to be induced by insulin signaling [51], and is linked through BC021367 (a transmembrane protein also known as Tmem161a) to the levels of free fatty acids in our model. We also see three genes implicated in hypertension: Wisp1, Gna14, and F11r. The gene WNT1 induced signaling pathway protein 1 (Wisp1) is connected with HDL levels through the N-myc downstream regulated gene 1 (Ndrg1) gene in our model. In addition, guanine nucleotide binding protein, alpha 14 (Gna14) is directly linked to weight. Finally, the gene F11r, also known as junctional adhesion molecule-1 (JAM-1) is related to both the total cholesterol levels in our model, as well as the combined LDL and VLDL cholesterol levels, through the 1190002J23Ri expression probe i.e. kelch domain containing 9 (Klhdc9) gene.

We also observe previously identified obesity associated genes, such as Zfp69, Slc24a3, Qpctl, Atp10a, and Folr2. This coverage of a broad spectrum of previously identified etiologies underlying obesity indicates the quality of the data as well as the predictions, given the complex nature of the obesity phenotype. In addition, through the network construction we were able to generate novel predictions, such as a *cis*-eQTL near rs3686646 may interact with Cytochrome c assembly, which in turn may have an impact on weight. In addition, we predict that the gene Slc24a3 modulates the effect of Crhr1 and Qpctl on the levels of glucose in the blood.

These additional network inferences also provide information with respect to how the effect of a given gene on a downstream metabolic phenotype may be mediated (Figure 5). For example, solute carrier family 24, member 3 (Slc24a3), which has been previously identified as having significantly decreased expression in diet-sensitive obese women and is directly linked to glucose levels in our network [52], has both the genes corticotropin releasing hormone receptor 1 (Crhr1) and glutaminyl-peptide cyclotransferase-like (Qpctl) directly linked to it. Both of these genes have been previously implicated as candidate obesity genes [50,53]. This suggests that the effect of the obesity risk associated with Qpctl andCrhr1 are mediated by Slc24a3's effect on the levels of glucose in the bloodstream.

The network connections recovered by our method identified a number of novel features important for obesity related phenotypes not previously identified by network analysis of these data. This includes the zinc-fingered protein 69 (Zfp69), which is directly linked to weight in our model. This gene has previously been identified as a candidate gene, for the diabetogenic effect of the Nidd/SJL loci in obese mice [54]. Another variable is the expression of cannabinoid receptor 2 (Cnr2), which is connected to both free fatty acids and glucose in our model, and that has been shown to mediate an innate immune response leading to inflammation in obese mouse adipocytes [49].

## Methods

### The network model

Similarly to Yin and Li [55], we assume that the expression data for the *i^th ^*sample (**y_i_**) conditioned on a set of genotypes and unpenalized covariates (**x_i_**) is distributed normally $y_{i}|x_{i}^{iid}\sim N\left(\Gamma x_{i},\Theta_{yy}^{-1}\right)$, with means determined by possibly sparse linear functions of genotypes and unpenalized covariates (Γ**x_i_**) as well as a sparse precision matrix (Θ**_yy_**) (i.e. the inverse covariance matrix). Yet, in contrast to Yin and Li [55], we define an alternative parameterization of the mean effects Γ, such that $\Gamma =\Theta_{yx}^{T}\Theta_{yy}^{-1}$. This lets us consider not only the conditional independencies among phenotypes correcting for the effect of genotypes and covariates as in Yin and Li [55], but this also allows us to identify a set of genotypic effects $\left(\Theta_{yx}^{T}\right)$ that directly takes into account the conditional independence structure among the expression phenotypes. The log-likelihood defined by this model is as follows [55]:

(1) $$\text{log}\left(Y|X,\Theta \right)\propto \text{log}\left\{\text{det}\left(\Theta_{yy}\right)\right\}-\text{Tr}\left(S\Theta \right),$$

where:

(2) $$\Theta =\left[\begin{array}{cc} \Theta_{yy} & \Theta_{yx}\\ \Theta_{yx}^{T} & \Theta_{xx} \end{array}\right],$$

and

(3) $$S=\frac{1}{n}\left[\begin{array}{cc} Y^{T}Y & Y^{T}X\\ X^{T}Y & X^{T}X \end{array}\right],$$

being the sample covariance matrix, **X **and **Y **mean-centered, **X **being an *n *× *m *matrix of genotypes and fixed effects, and **Y **being an *n *× *p *matrix of expression or downstream phenotypes. The elements *θ_ij _*of the matrix Θ represent the pairwise Markov dependencies of the random variables **Y **[34]. Intuitively, the set of non-zero *θ_ij _*parameters for a given random variable *y_i_*, defines the set of other phenotypes once conditioned on, make *y_i _*probabilistically independent from the rest of the variables in the model (also known as the neighborhood of *y_i_*). In this model everything is conditional on the state of the entire set of genotypes and fixed effects. The non-zero structure of the Θ**_yy _**sub-matrix specifies a conditional Markov random field among the expression or downstream phenotypes. Accordingly, the element $\theta_{yy}^{ij}$ for *i *≠ *j *of the Θ**_yy _**matrix is zero iff

(4) $$p\left(y_{i},y_{j}|Y_{-\left(i,j\right)},X\right)=p\left(y_{i}|Y_{-\left(i,j\right)},X\right)p\left(y_{j}|Y_{-\left(i,j\right)},X\right),$$

i.e. the probability distribution satisfies the local Markov property [34] with respect to an undirected graph $G=\left(\text{V},\text{E}\right)$, with **Y**_-(*i*, *j*) _indicating the set of other phenotypes, excluding the single variables *y_i _*and *y_j_*. Since this is a Markov random field conditioned on **X **assuming an underlying linear model, the non-zero structure of the Θ**_xy _**sub-matrix does not imply a factorization over an underlying probability density, but the element $\theta_{xy}^{ij}$ is zero iff

(5) $$p\left(y_{j}|X_{-i},Y_{-j},x_{i}\right)=p\left(y_{j}|X_{-i},Y_{-j}\right),$$

i.e. the conditional distribution of *y_j _*is the same, when conditioning on **X**_-**i **_and **Y**_-**j**_, whether one conditions on *x_i _*or not. Finally, since this is a conditional Markov random field, the rank of the matrix Θ is *p *and $\Theta_{xx}=\Theta_{xy}\Theta_{yy}^{-1}\Theta_{yx}$.

### Undirected network inference

To infer the structure of the underlying undirected graph, many authors have proposed putting different forms of element-wise penalties on the Θ matrix, such as the lasso (*l*_1 _norm) [12,56]. Additionally, as other authors have noted [57], the positive-semi definite constraint on Θ imposed by the log{det} function in the log likelihood makes optimization of the full likelihood problem challenging for large scale problems, especially when the number of phenotypes and genotypes *p *+ *m *greatly exceeds the sample size *n*. Therefore, instead of solving the full likelihood optimization problem, we follow the general strategy of Meinshausen and Bühlmann, Zhou et al., and Kraemer et al. [12,13,22], and treat the structure learning problem as a neighborhood identification problem; i.e. we perform model selection on a set of uncoupled regression equations, where each expression phenotype is regressed on every other phenotype, and genotype. At the end of this process we resolve the neighborhoods of each gene expression product by averaging the posterior probabilities of edge inclusion in both directions of regression.

We define a given multiple regression equation as:

(6) $$y_{i}=\mu +\overset{p-1}{\underset{j}{\sum }}\;z_{ij}\beta_{j}^{y}+\overset{m}{\underset{l}{\sum }}\;x_{il}\beta_{l}^{x}+\overset{l}{\underset{k}{\sum }}\;t_{ik}\alpha_{k}+e_{i},$$

where *y_i _*is *i^th ^*sample of a given phenotype, the population mean is modeled as a fixed effect *μ*, *z_ij _*is the *i^th ^*sample of the *j^th ^*phenotype, excluding the phenotype $y,\beta_{j}^{y}$ is the effect of the *j^th ^*phenotype, *x_il _*is the *i^th ^*sample of the *l^th ^*genotype, $\beta_{l}^{x}$ is the effect of the *l^th ^*genotype, *t_ik _*is the *i^th ^*sample of the *k^th ^*non-penalized effect, *α_k _*is the effect of this *k^th ^*feature, and *e^i ^*is the residual error term, assumed to be normally distributed with mean zero, and variance $\sigma_{e}^{2}$. In general we include the top 20 eigenvectors from a principal component analysis of the expression phenotypes as unpenalized covariates *t_ik _*for each penalized regression model.

### Connection between penalized regression solutions and the network model

While the likelihood defined in Equation 1 corresponds to a conditional GGM corresponding to the joint distribution of the gene expression phenotypes (**Y**) conditional on some set of genotypes and fixed effects (**X**), our approach focuses on solving a set of penalized regression equations for each phenotype (as in Equation 6). Our assumption (as in Meinshausen and Bühlmann [12]) is that the set of variables that are selected in a particular regression model for a given phenotype (e.g. which $\beta_{j}^{y}$ and $\beta_{l}^{x}$ are non-zero will exactly specify which set of elements of Θ are non-zero). For example, if in the penalized regression model for the 5*^th ^*phenotype we find that $\beta_{1}^{y}$, $\beta_{3}^{y}$, and $\beta_{4}^{x}$ are non-zero, then this would indicate that the corresponding elements of Θ, $\theta_{yy}^{15}$, $\theta_{yy}^{35}$, and $\theta_{yx}^{54}$ would be non-zero, and the corresponding conditional independence properties implied by Equation (4) and Equation (5) would be true for these variables.

### Bayesian hierarchical model for sparse feature selection

Given the regression equation defined in Equation 6, we define the following hierarchical model, similar in vein to Zhang et al. and Logsdon et al. [25,29]:

(7) $$\beta_{j}\sim p_{\beta =0}\text{I}\left[\beta =0\right]+p_{\beta \neq 0}N\left(0,\sigma_{\beta }^{2}\right),$$

(8) $$p_{\beta =0},p_{\beta \neq 0}\sim \text{Beta}\left(1,1\right),$$

(9) $$\sigma_{\beta }^{-2}\sim \Gamma \left(2,1/2\right),$$

(10) $$\sigma_{e}^{-2}\sim \Gamma \left(2,1/2\right),$$

with the additional truncation restriction on the prior distribution over *p*_*β *≠ 0 _of $p_{\beta \neq 0}\leq \sqrt{n}/\left(m+p-1\right)$.

This mixture penalty in a Bayesian framework has attractive theoretical properties, including bounded shrinkage and indications that it may approach optimal efficiency for sparse underlying parameter spaces [58] and may still be model selection consistent when the irrepresentability condition is not met [27]. One of the main advantages of this approach is that the hierarchical model can adaptively shrink the penalty to match the sparsity of the underlying parameter space, without having to resort to prediction based metrics like cross-validation which can overestimate model size or possibly heuristic model complexity measures based on information criterion such as Akaike's Information Criterion (AIC) or Bayesian Information Criterion (BIC).

Because the mixture penalty is non-convex, the posterior surface can be highly multi-modal and each mode in the posterior density can represent a different set of identified features (i.e. neighborhood). A well characterized weakness of the *l*_0 _type penalty (i.e. best subset selection) is the instability of the identified solutions [59]. One of the most important novel contributions of our algorithm is a Bayesian model averaging step [60]. We perform Bayesian model averaging across the identified modes by re-weighting the posterior probability of inclusion for each feature (${\hat{p}}_{j}$), proportional to the estimated volume underneath each identified mode (a measure of the relative evidence of a given model) based on the lower bound, an approach similar to bagging [61]. Because the algorithm is very fast, we can run it many times (up to thousands) and identify many models, along with the relative evidence of each model identified, based on the lower bound (Equation 17 in the Supporting Information), and integrate the evidence across the models. This approach is effective at integrating out model uncertainty, and generating the best estimates of which interactions are most strongly supported by the data.

### Variational spike-and-slab algorithm

The variational Bayes approximation consists of minimizing the Kullback-Leibler divergence between an approximate factorized posterior distribution $q_{\beta_{1}}\left(\beta_{1}\right)\cdots q_{\beta_{m}}\left(\beta_{m}\right)q_{p_{\beta \neq 0}}\left(p_{\beta \neq 0}\right)q_{\sigma_{e}}\left(\sigma_{e}\right)q_{\sigma_{\beta }}\left(\sigma_{\beta }\right)$ and the full posterior distribution *p*(*β*_1_,...,*β_m_*, *p*_*β *≠ 0_, *σ_e_*, *σ_β_*), using iterative expectation type steps as in an Expectation-Maximization algorithm [38,39]. Given this optimization procedure, the variational Bayes distributional approximation for the posterior distribution of an arbitrary parameter *θ *(i.e. *β*_1_,...,*β*_*p *+ *m*-1_, *σ_e_*, *σ_β_*, *p*_*β *≠ 0_) is given as follows:

(11) $$q_{\theta_{j}}^{t+1}\left(\theta_{j}\right)=\frac{1}{Z_{\theta_{j}}}\text{exp}\left\{\int q_{\theta_{-j}}^{t}\left(\theta_{-j}\right)d\theta_{-j}\text{log}\left\{p\left(\theta |y\right)\right\}\right\},$$

where a factorization is defined over the joint approximate posterior distribution of parameters:

(12) $$q_{\theta }\left(\theta \right)=\underset{i}{\prod }\;q_{\theta_{i}}\left(\theta_{i}\right),$$

and the integral in Equation 11 at iteration *t *is taken with respect to every approximate distribution except $q_{\theta_{j}}^{t}\left(\theta_{j}\right)$. The posterior density *p*(*θ*|**y**) in Equation 11 is defined based on multiplying the likelihood for the model in Equation 6 with the priors in Equation 7-10. The details of this approximation for each density are presented in the Additional file 1. This factorization is required to solve for closed form iterative updates associated with the spike-and-slab prior distribution. A probability of inclusion statistic, *p_j _*is computed after the algorithm converges, and this statistic is averaged across all models identified (i.e. modes in the posterior surface), based on the total evidence for each model (i.e. Equation 17 in the Supporting Information). This model averaged probability of inclusion statistic, ${\hat{p}}_{j}$ is used to determine whether the *j^th ^*feature is included in the model, at a given threshold.

### Lasso with type I error control

We compared our variational spike-and-slab algorithm with two alternative methods proposed to bound the number of type I errors when using lasso penalized regression. The first method we compared was the randomized lasso with stability selection, as described by Meinshausen and Bühlmann [40]. As with our method, we performed this approach by solving a penalized regression model for each phenotype in the network individually, then afterwards we compared different methods for combining the results. As in Meinshausen and Bühlmann [40], we implemented the randomized lasso with sampling weights for the variables in the regression sampled from a Unif(0.2, 1.0), then we performed the stability selection procedure by sampling *n*/2 observations, and running 100 replicate instances of this two-level randomized algorithm. This was run using the glmnet package in R [62] on a grid of 100 logarithmically spaced penalty parameters *λ *= {10^-2^,...,10^2^} (this range of penalization was sufficient such that the approach never chose a level of penalization on the boundaries). As in Meinshausen and Bühlmann [40], we focused on bounding the number of type I errors by choosing an empirical probability of selection cutoff of 0.9, then choosing the level of penalization that returns the expected number of selected variables which satisfies the bound on the number of false positives [40]. We looked at bounds of both 1 and 1000 expected false positives for the entire network, to explore both conservative and liberal choices of the cutoffs.

The alternative approach we used to bound the number of type I errors was based on the original Meinshausen and Bühlmann network algorithm [12], where they bound the number of type I errors between connectivity components in a graph. Intuitively, a connectivity component is just the set of variables for any given variable that can be reached through some path within the graph. We used their choice of penalty parameter $\lambda \left(\alpha \right)=2\frac{\hat{\sigma }}{\sqrt{n}}{\tilde{\Phi }}^{-1}\left(\frac{\alpha }{2p^{2}}\right)$, where *α *is the probability of making a type I error, $\hat{\sigma }=n^{-1}\sum_{i}\;y_{i}^{2}$ for any given phenotype *y*, *p *is the number of variables in the model, and ${\tilde{\Phi }}^{-1}=1-\Phi$ (with Φ the c.d.f. of the standard normal density function). We investigated bounds of both 1 and 1000 expected false positives for the entire network, by running the regular lasso penalized regression method with the glmnet package in R [62] and this level of penalization.

### Simulations

For the simulations depicted in Figures 1, 2, we simulated twenty replicate networks through the following procedure: first, a random directed graph with *p *= 1000 variables and adjacency matrix **A **was generated by sampling edges between variables with probability 1/*p *= 10^-3 ^for all *p*(*p*-1) edges. Next, each edge was weighted by a $N\left(0,1\right)$ random variable. Third, the diagonal of **A **was set to one. Finally, we constructed the precision matrix Θ = **AA^T ^**(i.e. inverse covariance matrix), and sampled a data-set **Y **from the multivariate normal distribution $N\left(0,\Theta^{-1}\right)$, with 300 independent observations. Because of the moralization (i.e. edges induced between nodes that share parents [39]) produced by converting the directed graph **A **into an undirected graph Θ, the average number of edges per node was 1.47 instead of ≈1. It is important to note that we did not include any unpenalized covariates or genotypes in this particular simulation. Additional algorithms used for comparison with analysis of further simulated data are shown in the Additional file 1.

### Data analysis

We analyzed the F_2 _progeny of a cross between the C57BL/6 J (B6) and C3H/HeJ (C3H) strains on an apolipoprotein E null (ApoE -/-) background (BXH.ApoE^-/-^) [9,31]. After further filtering the data to a set of shared samples across all variables, we were left with 298 individuals, 22 downstream phenotypes, 1,347 genetic markers, and 23,574 expression probes. We included sex as well as the 20 first eigenvectors from a principal component analysis computed across samples for expression phenotypes as fixed effects, and ran the first phase of the algorithm (i.e. feature selection on the downstream phenotypes) with 1,000 random restarts of the algorithm to get good coverage of the posterior probability surface associated with model uncertainty for each downstream phenotype. We then ran the second phase of the algorithm between just expression traits and genotypes, still incorporating the 20 eigenvectors from principal component analysis and sex as fixed effects, with 50 random restarts. All network diagrams in Figures 3, 4, 5 were generated with the network package in R [63].

## Authors' contributions

BAL derived and implemented the algorithm, and performed the simulations and data analysis. BAL and JGM designed the simulations and data analysis. GEH implemented and ran the lasso algorithm for the data analysis. BAL and JGM wrote the manuscript. All authors read and approved the final version of this manuscript.

## Supplementary Material

Additional file 1 **Supplementary methods and results**. A supplementary file containing additional descriptions of the variational method, and additional results from simulations and data analysis. Click here for file
