# Supplementary material for: Mouse obesity network reconstruction with a variational Bayes algorithm to employ aggressive false positive control
Source: BMC Bioinformatics. 2012 Apr 2;13:53. doi: 10.1186/1471-2105-13-53 (PMC3338387; doi:10.1186/1471-2105-13-53)
Supplement: Additional file 1 — Supplementary methods and results. A supplementary file containing additional descriptions of the variational method, and additional results from simulations and data analysis. [file 1471-2105-13-53-S1.PDF]

Supporting Information Text

Variational spike and slab updates

The variational Bayes expectation (VBE) steps are defined as:

$$q^{t+1}_{\beta_j}\left(\beta_j\right) = (1 - p^t_j)\mathrm{I}\left[\beta_j = 0\right] + p^t_j \mathrm{N}\left(\mu^t_j, \sigma^{2\,t}_j\right) \tag{1}$$

with sufficient statistics:

$$\begin{aligned}
\mu^t_j &= \frac{\sum_{i=1}^n z_{ij}\langle r_{-j}\rangle}{\sum_{i=1}^n z^2_{ij} + \langle\sigma^{-2}_\beta\rangle/\langle\sigma^{-2}_e\rangle},\\
\sigma^{-2\,t}_j &= \langle\sigma^{-2}_e\rangle\sum_{i=1}^n z^2_{ij} + \langle\sigma^{-2}_\beta\rangle,\\
p^t_j &= \frac{1}{1 + C^t_j},
\end{aligned} \tag{2}$$

and expectations:

$$\begin{aligned}
\langle\beta_j\rangle &= p^t_j\mu^t_j,\\
\langle\beta^2_j\rangle &= p^t_j\left(\mu^{2\,t}_j + \sigma^{2\,t}_j\right),
\end{aligned} \tag{3}$$

where $\langle r_{-j}\rangle$ is the expectation of the linear residual term with respect to each approximate distribution, and

$$\begin{aligned}
C^t_j = &\left(1 - \exp\left\{\langle\log\left(p_{\beta\neq 0}\right)\rangle\right\}\right)/\\
&\left(2\sigma^t_j \exp\left\{\frac{1}{2}\left(2\langle\log\left(p_{\beta\neq 0}\right)\rangle + \mu^{2\,t}_j/\left(\sigma^{2\,t}_j\right) + \langle\log\left(\sigma^{-2}_\beta\right)\rangle\right)\right\}\right).
\end{aligned} \tag{4}$$

In this case, z_{ij} is a ith sample of the jth penalized phenotype or genotype. The update for the distribution of the approximate error variance is

$$q^{t+1}_{\sigma^{-2}_e}\left(\sigma^{-2}_e\right) = \Gamma\left(\eta_1, \eta_2\right), \tag{5}$$

with sufficient statistics:

$$\begin{aligned}
\eta_1 &= \frac{n+1}{2}\\
\eta_2 &= \frac{\langle U\rangle + 1}{2}
\end{aligned} \tag{6}$$

with expectations:

$$\begin{aligned}
\langle\sigma^{-2}_e\rangle &= \frac{\eta_1}{\eta_2}\\
\langle\log\left(\sigma^{-2}_e\right)\rangle &= \psi\left(\eta_1\right) + \log\left(1/\eta_2\right)
\end{aligned} \tag{7}$$

with $\langle U\rangle$ the expectation of the residual sum of square errors with respect to each current approximating distribution, and $\psi(x)$ the digamma function.

$$q^{t+1}_{\sigma_\beta^{-2}}\left(\sigma_\beta^{-2}\right) = \Gamma\left(\zeta_1, \zeta_2\right), \tag{8}$$

with sufficient statistics:

$$\zeta_1 = \frac{\sum p_j^t + 1}{2},$$
$$\zeta_2 = \frac{\sum\langle\beta_j^2\rangle + 1}{2}, \tag{9}$$

with expectations:

$$\langle\sigma_\beta^{-2}\rangle = \frac{\zeta_1}{\zeta_2},$$
$$\langle\log\left(\sigma_\beta^{-2}\right)\rangle = \psi\left(\zeta_1\right) + \log\left(1/\zeta_2\right), \tag{10}$$

$$q^{t+1}_{p_{\beta\neq0}}\left(p_{\beta\neq0}, 1 - p_{\beta\neq0}\right) = \text{TBeta}\left(\rho_1, \rho_2, \frac{\sqrt{n}}{m+p-1}\right) \tag{11}$$

with sufficient statistics:

$$\rho_1 = \sum p_j^t + 1$$

,

$$\rho_2 = m - \sum p_j^t + 1, \tag{12}$$

and expectations $\langle p_{\beta\neq0}\rangle$ and $\langle\log\left(p_{\beta\neq0}\right)\rangle$ computed by numerical integration (with $\text{TBeta}\left(x,y,z\right)$ being a truncated Beta distribution with parameter x, y and support on $[0, z]$). A maximization step of the effect α of the fixed covariates, $\mathbf{T}$, is defined as:

$$\hat{\alpha}^{t+1} = \left(\mathbf{T}'\mathbf{W}\mathbf{T}\right)^{-1}\mathbf{T}'\mathbf{W}\left(\mathbf{y} - \mathbf{X}\langle\beta\rangle\right) \tag{13}$$

with $\mathbf{W} = \text{diag}\left(\langle\sigma_e^{-2}\rangle\right)$. The lower bound:

$$\mathcal{L}^{t+1} = -\frac{\sigma_e^{-2}}{2}\langle U\rangle + \langle\theta\rangle, \tag{14}$$

is computed every iteration, and stored after the algorithm converges (in practice when the per iteration change $\Delta\mathcal{L} \leq 0.0001$), where

$$\langle\theta\rangle = \rho_2\log\left(1 - \exp\left\{\langle\log\left(p_{\beta\neq0}\right)\rangle\right\}\right) +$$
$$\frac{1}{2}\rho_1\left(\langle\log\left(\sigma_\beta^{-2}\right)\rangle + 1 + 2\langle\log\left(p_{\beta\neq0}\right)\rangle\right) -$$

$$\zeta_2\left(\langle\sigma_\beta^{-2}\rangle+1\right)+\frac{1}{2}\left(n+1\right)\langle\log\left(\sigma_e^{-2}\right)\rangle. \tag{15}$$

Multiple re-orderings of all variables are initialized so as to identify multiple modes in the approximate posterior surface. We initialize all β parameters to zero, and only change the order in which we update the β parameters for different restarts of the algorithm. We assume that each unique mode in the approximate posterior surface corresponds to a unique model with an associated posterior probability. We generate an approximation of this posterior probability of a given model by assigning the following probabilities:

$$p\left(M_i\right)=\frac{\exp\left(\mathcal{L}_i\right)}{\sum\exp\left(\mathcal{L}_i\right)}. \tag{16}$$

Approximate Bayesian model averaged results are generated for all the sufficient statistics/expectations, including the posterior probability of inclusion in the model, p_j:

$$\hat{p}_j=\sum_i p_{ji}p\left(M_i\right) \tag{17}$$

If there is a high degree of model uncertainty in the estimate, then Bayesian model averaging will reduce the variance of estimates. Before we run the algorithm, we rescale and recenter all the features to have mean zero, and variance one, so as not to penalize each possible feature (i.e. expression trait of genetic marker) differently based on the scale of the feature. We also set sporadic missing data to the empirical mean of the observed data for all variables. We include a weak pre-filtering step, where we only include the genetic markers and gene expression phenotypes which have $P<0.1$ from a marginal test in a linear model (conditioned on the fixed effects, $\mathbf{T}$). This is motivated by recent theoretical work by Fan et al. [1], that suggests that a simple marginal test statistic can be used to effectively screen features that are not relevant, i.e. not in the full model. The size of the reduced filtered set of features is used when computing the truncation of the distribution over $p_{\beta\neq 0}$. This pre-filtering step was also included for the lasso methods with stability selection, stringent penalization, and cross-validation when analyzing the downstream phenotype data. Alternatively, this pre-filtering step was not included in the simulations in the main text of the paper (e.g. the simulations for Figure 1 and Figure 2) for either variational spike and slab or lasso methods. While for the neutral expectation, if all variables being tested are independent of the dependent variable then a filter of $P<0.1$ would remove 90% of the variables, in the data analysis we observed for most downstream and expression phenotypes on average only $\approx 70\%$ of the variables were removed (so ≈ 8000 features included) for any particular regression model. This is likely because of the rich correlation structure within the data-set.

Lasso and Adaptive lasso

First proposed by Tibshirani [2], the lasso, and the adaptive lasso [3], were implemented in C++ using a very efficient cyclic coordinate descent algorithm (Friedman et al, 2007) to solve the penalized regression problem:

$$\arg\max_{\beta}\{-\sum_{i=1}^{n}(y_i - z_i\beta)^2 - \lambda \sum_{j=1}^{p+m-1} |\beta_j|\} \tag{18}$$

and then using the coefficients from this problem to solve the following adaptive lasso problem:

$$\arg\max_{\zeta}\{-\sum_{i=1}^{n}(y_i - z_i\zeta)^2 - \lambda \sum_{j=1}^{p+m-1} w_j|\zeta_j|\} \tag{19}$$

where $w_j = |\hat{\beta}_j|^{-1/2}$ is a variable-specific weight learned from the original lasso optimization, z is the combined gene expression products and genetic marker genotypes, β and ζ are vectors of the corresponding regression coefficients. The hyperparameter λ determines the strength of the penalty on the magnitude of the regression coefficients and was determined by 10-fold cross-validation. Unpenalized variables are easily accommodated in z by setting the penalty to zero for relevant variables.

Shrinkage estimator

The shrinkage estimator of Schäfer and Strimmer [4] involves taking a convex combination of a unregularized estimate of the sample covariance matrix, $\mathbf{\Sigma}$, and combines it with a low-rank regularized estimate, $\mathbf{T}$, with a weighting parameter λ. The inverse covariance matrix is inferred based on this regularized estimate of the sample covariance. The number of significant non-zero parameter is determined based on an empirical estimation of the false discovery rate [4].

$$\hat{\mathbf{\Sigma}}_{\lambda} = \lambda\hat{\mathbf{T}} + (1-\lambda)\,\hat{\mathbf{\Sigma}} \tag{20}$$

Partial least squares estimator

The partial least squares estimator in regularized regression was proposed by Tenenahaus et al. [5], and is defined as a constrained optimization problem for identifying a small set of orthogonal predictors, with maximal covariance with the response variable in question. The regularization from this approach arises by choosing a small subset of orthogonal predictors, where the number of predictors is chosen by cross-validation [6].

Ridge estimator

As opposed to an l_1 lasso penalty, Kraemer et al. [6] propose a ridge penalty (i.e. an l_2^2 penalty), and use an empirical control of false discovery rate (FDR) based on semi-parametric estimation of either the tail area-based FDR or local FDR [7]:

$$\arg\max_{\beta} \left\{ -\sum_{i=1}^{n} (y_i - z_i \beta)^2 - \lambda \sum_{j=1}^{p+m-1} \beta_j^2 \right\} \tag{21}$$

Simulation analyses and comparison to other network recovery algorithms

To test our method on simulated data, we compared it to a set of other methods that were recently proposed and combined [6] for sparse, regularized undirected network inference. To allow a consistent comparison with Kraemer et al., we use the same set of simulation parameters, network connectivity, and implementation of the five methods, which included ridge regularized regression, shrinkage estimation, partial least squares regression, along with the lasso and the adaptive lasso. In this case we simulated data from a network of 100 gene expression phenotypes (with no genotypes), with a density of 0.05 (i.e. 248 undirected edges were randomly assigned between pairs of variables, across 10 replicate simulations). A constant variance of one is assumed across all phenotypes, and the edges weights are simulated as uniformly distributed between -1 and 1; and the full $\mathbf{\Theta_{yy}}$ matrix is rescaled such that $\text{diag}(\mathbf{\Theta_{yy}}) = 1$. The performance of all the methods for varying sample sizes is illustrated in Figure S4-S6 in terms of a precision-recall curve in terms of the combined estimated regression coefficients for each method (i.e. the β coefficients). We see that the three best methods are the adaptive lasso, the lasso, and the variational spike and slab algorithm, which all appear to be close in terms of performance for the range of high true discovery rate (TDR or precision), for similar power (recall).

While this performance is similar among these three methods as a function of the estimated regression coefficient, there is still a fundamental problem associated with how to decide which nonzero elements are statistically significantly different from zero, where the null distribution of any test statistic would be non-trivial [8]. In Figure S7 we see the true discovery rate for different sample sizes in terms of the features that are very confidently returned by the variational spike and slab, the lasso and the adaptive lasso algorithms. For the variational spike and slab algorithm, we see that if we choose only the edges which have posterior probability, $\hat{p}_j > 0.99$, we can aggressively control the FDR. Whereas, if we include every feature that is proposed by either the lasso or the adaptive lasso, we have a significant number of false

positives that get carried along. This suggests that we can use the variational spike and slab to identify edges between genes only when there is very strong statistical support for the interaction in the data. It also suggests that the variational spike and slab algorithm does a much better job at estimating an appropriate level of penalization to restrict the number of false positives within the model.

References

1. Fan J, Lv J: **Sure independence screening for ultrahigh dimensional feature space**. *J. Roy. Stat. Soc. B. Met.* 2008, **70**(5):849–911.
2. Tibshirani R: **Regression shrinkage and selection via the lasso**. *J. Roy. Stat. Soc. B. Met.* 1996, **58**:267–288.
3. Zou H: **The adaptive lasso and its oracle properties**. *J. Am. Stat. Assoc.* 2006, **101**(476):1418–1429.
4. Schafer J, Strimmer K: **An empirical Bayes approach to inferring large-scale gene association networks**. *Bioinformatics* 2005, **21**(6):754–764.
5. Tenenhaus A, Guillemot V, Gidrol X, Frouin V: **Gene association networks from microarray data using a regularized estimation of partial correlation based on PLS regression**. *IEEE IEEE/ACM Transactions on Computational Biology and Bioinformatics* 2008.
6. Kraemer N, Schafer J, Boulesteix A: **Regularized estimation of large-scale gene association networks using graphical Gaussian models**. *BMC Bioinformatics* 2009, **10**:384.
7. Strimmer K: **A unified approach to false discovery rate estimation**. *BMC Bioinformatics* 2008, **9**:303.
8. Wu T, Chen Y, Hastie T, Sobel E, Lange K: **Genome-wide association analysis by lasso penalized logistic regression**. *Bioinformatics* 2009, **25**(6):714.
9. Scherneck S, Nestler M, Vogel H, Blüher M, Block M, Diaz M, Herzig S, Schulz N, Teichert M, Tischer S, et al.: **Positional cloning of zinc finger domain transcription factor Zfp69, a candidate gene for obesity-associated diabetes contributed by mouse locus Nidd/SJL**. *PLoS Genet.* 2009, **5**(7):593–596.
10. Kohara K, Tabara Y, Nakura J, Imai Y, Ohkubo T, Hata A, Soma M, Nakayama T, Umemura S, Hirawa N, et al.: **Identification of hypertension-susceptibility genes and pathways by a systemic multiple candidate gene approach: the millennium genome project for hypertension**. *Hypertens. Res.* 2008, **31**(2):203–212.
11. Waki H, Liu B, Miyake M, Katahira K, Murphy D, Kasparov S, Paton J: **Junctional adhesion molecule-1 is upregulated in spontaneously hypertensive rats: evidence for a prohypertensive role within the brain stem**. *Hypertension* 2007, **49**(6):1321.
12. Liu H, Han J, Cao S, Hong T, Zhuo D, Shi J, Liu Z, Cao W: **Hepatic autophagy is suppressed in the presence of insulin resistance and hyperinsulinemia**. *J. Biol. Chem.* 2009, **284**(45):31484.
13. Yamada Y, Ando F, Shimokata H: **Association of polymorphisms of SORBS1, GCK and WISP1 with hypertension in community-dwelling Japanese individuals**. *Hypertens. Res.* 2009, **32**(5):325–331.
14. Kim S, Sohn I, Ahn J, Lee K, Lee Y, Lee Y: **Hepatic gene expression profiles in a long-term high-fat diet-induced obesity mouse model**. *Gene* 2004, **340**:99–109.
15. Peltola P, Pihlajamäki J, Koutnikova H, Ruotsalainen E, Salmenniemi U, Vauhkonen I, Kainulainen S, Gylling H, Miettinen T, Auwerx J, et al.: **Visceral Obesity is Associated with High Levels of Serum Squalene**. *Obesity* 2006, **14**(7):1155–1163.
16. Keeton A, Amsler M, Venable D, Messina J: **Insulin signal transduction pathways and insulin-induced gene expression**. *J. Biol. Chem.* 2002, **277**(50):48565.
17. Gerrits M, Ghosh S, Kavaslar N, Hill B, Tour A, Seifert E, Beauchamp B, Gorman S, Stuart J, Dent R, et al.: **Distinct skeletal muscle fiber characteristics and gene expression in diet-sensitive versus diet-resistant obesity**. *J. Lipid. Res.* 2010, **51**(8):2394.
18. Cone R: **Editorial: The Corticotropin-Releasing Hormone System and Feeding Behavior–A Complex Web Begins to Unravel**. *Endocrinology* 2000, **141**(8):2713.
19. Perusse L, Rankinen T, Zuberi A, Chagnon Y, Weisnagel S, Argyropoulos G, Walts B, Snyder E, Bouchard C: **The human obesity gene map: the 2004 update**. *Obesity* 2005, **13**(3):381–490.
20. Speliotes E, Willer C, Berndt S, Monda K, Thorleifsson G, Jackson A, Allen H, Lindgren C, Luan J, M "agi R, et al.: **Association analyses of 249,796 individuals reveal 18 new loci associated with body mass index**. *Nat. Genet.* 2010.
21. Davies M, Gordon J, Gearing A, Pigott R, Woolf N, Katz D, Kyriakopoulos A: **The expression of the adhesion molecules ICAM-1, VCAM-1, PECAM, and E-selectin in human atherosclerosis**. *J. Pathol.* 1993, **171**(3):223–229.

22. Otsuki M, Hashimoto K, Morimoto Y, Kishimoto T, Kasayama S: **Circulating vascular cell adhesion molecule-1 (VCAM-1) in atherosclerotic NIDDM patients.** *Diabetes* 1997, **46**(12):2096.

23. Bouchard C, Rankinen T, Chagnon Y, Rice T, Perusse L, Gagnon J, Borecki I, An P, Leon A, Skinner J, et al.: **Genomic scan for maximal oxygen uptake and its response to training in the HERITAGE Family Study***. *J. Appl. Physiol.* 2000, **88**(2):551.

24. Aulchenko Y, Pullen J, Kloosterman W, Yazdanpanah M, Hofman A, Vaessen N, Snijders P, Zubakov D, Mackay I, Olavesen M, et al.: **LPIN2 is associated with type 2 diabetes, glucose metabolism, and body composition**. *Diabetes* 2007, **56**(12):3020.

25. Klöting N, Klöting I: **Genetic variation in the multifunctional transcription factor Yy1 and type 1 diabetes mellitus in the BB rat**. *Mol. Genet. Metab.* 2004, **82**(3):255–259.

26. Zeyda M, Gollinger K, Kriehuber E, Kiefer F, Neuhofer A, Stulnig T: **Newly identified adipose tissue macrophage populations in obesity with distinct chemokine and chemokine receptor expression**. *Int. J. Obesity.* 2010.

27. Deveaux V, Cadoudal T, Ichigotani Y, Teixeira-Clerc F, Louvet A, Manin S, Nhieu J, Belot M, Zimmer A, Even P, et al.: **Cannabinoid CB2 receptor potentiates obesity-associated inflammation, insulin resistance and hepatic steatosis**. *PLoS One* 2009, **4**(6):e5844.

28. Dhar M, Webb L, Smith L, Hauser L, Johnson D, West D: **A novel ATPase on mouse chromosome 7 is a candidate gene for increased body fat**. *Physiol. Genomics.* 2000, **4**:93.

29. Gomez-Ambrosi J, Catalan V, Diez-Caballero A, Martinez-Cruz L, Gil M, Garcia-Foncillas J, Cienfuegos J, Salvador J, Mato J, Fruhbeck G: **Gene expression profile of omental adipose tissue in human obesity**. *FASEB. J.* 2003, :305911.

Supplementary Tables

Table S1 - Comparison of obesity phenotype analysis across methods

Model size, intersections, and proportion of significant associations based on an independently fit linear model, between the variational method (vb), the lasso (las), and the adaptive lasso (alas) where the lasso results were tuned using 10-fold cross-validation.

Pheno	vb	las	alas	vb ∩ las	vb ∩ alas	%P < 0.05 vb	%P < 0.05 las	%P < 0.05 alas
Weight	13	272	230	7	5	100%	14%	58%
Total Chol	12	224	173	7	5	100%	15%	42%
HDL	9	20	19	4	4	100%	50%	58%
UC	13	121	105	9	8	100%	22%	34%
FFA	9	258	214	5	4	100%	9%	43%
Glucose	8	214	173	5	4	100%	23%	46%
LDL+VLDL	12	219	178	5	5	100%	16%	34%

Table S2 - Obesity related interactions

Interactions identified by the variational method with previous evidence as being associated with obesity, or obesity related traits, with references.

Gene/SNP	Disease	Organism(s)	Reference
Zfp69	Candidate gene for diabetes associated with obesity	Mouse and Human	[9]
Gna14	Association study of hypertension	Human	[10]
F11r	Induces hypertension in the brain stem	Rat	[11]
Gabarapl1	Regulator of insulin dependent hepatic autophagy	Mouse	[12]
Wisp1	Association study of hypertension	Human	[13]
Fdft1	Squalene (cholesterol) biosynthesis gene	Mouse and Human	[14, 15]
Ier2	Induced gene in insulin signalling pathways	Rat	[16]
Slc24a3	Down regulated in diet sensitive obesity	Human	[17]
Crhr1	Candidate obesity gene possibly affecting feeding behavior	Mouse and Human	[18, 19]
Qpctl	Association study identified candidate obesity gene	Human	[20]
Vcam-1	Atherosclerotic plaque associated gene	Human	[21, 22]
Gch1	Identified in linkage studies of maximal sedentary oxygen uptake	Human	[23]
Dlgap1	Type-2 diabetes associated gene	Human	[24]
Yy1	Type-1 diabetes associated gene	Rat	[25]
Ccl19	Adipocyte inflammation	Human	[26]
Cnr2	Obesity associated adipocyte inflammation	Mouse	[27]
Atp10a/rs3664823	Obesity associated gene	Mouse	[28]
Folr2	Up-regulated in obesity associated adipose tissue	Human	[29]

Supplementary Figures

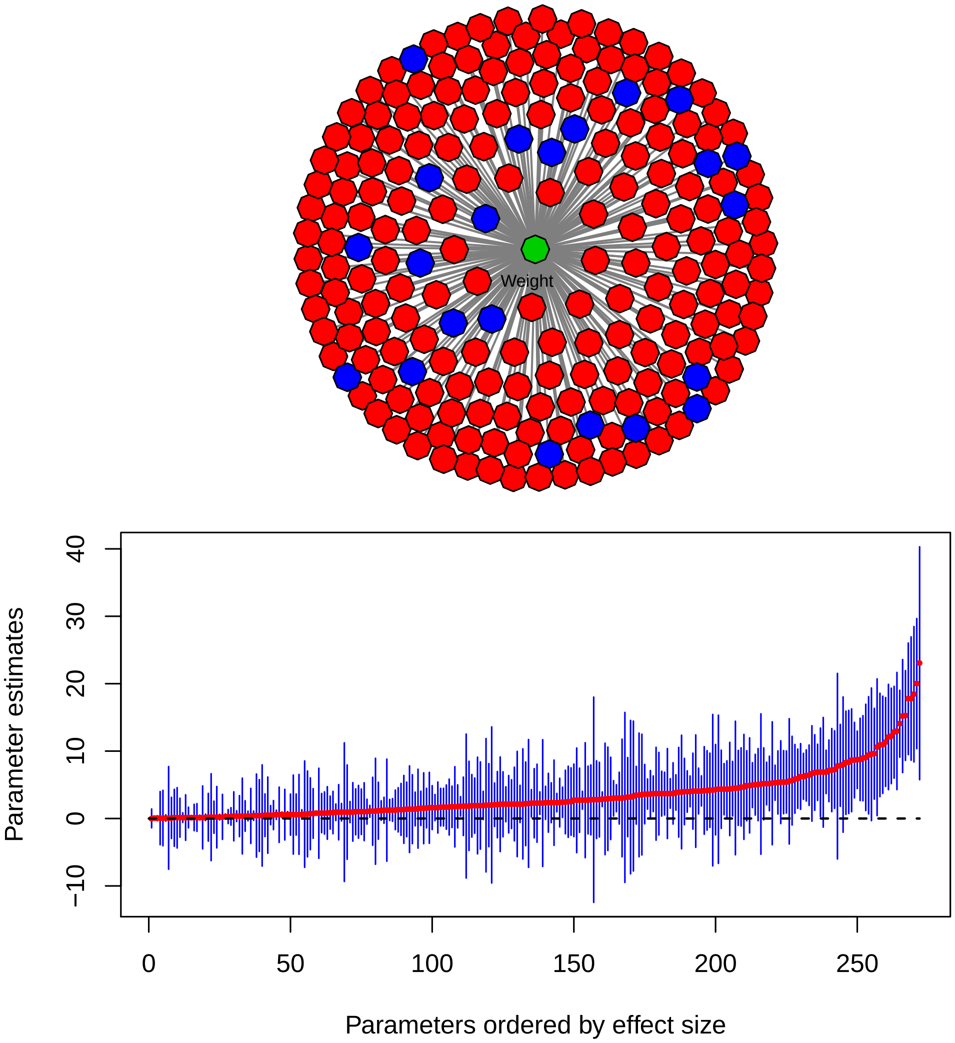

Figure S1 - Genetic markers and gene expression traits identified by the lasso with 10-fold cross-validation as being associated with weight in a mouse F_2 cross when analyzing an undirected graphical model. Top panel: Graphical depiction of relationship between the weight of the mouse (green), and the identified set of gene expression (red), and genetic marker interactions (blue). Bottom panel: Estimates in red and 95% confidence intervals (vertical blue lines) of the parameters describing the strength of regression relationship between each of the gene expression or genotype features with weight in an independently fit, unpenalized multiple regression model.

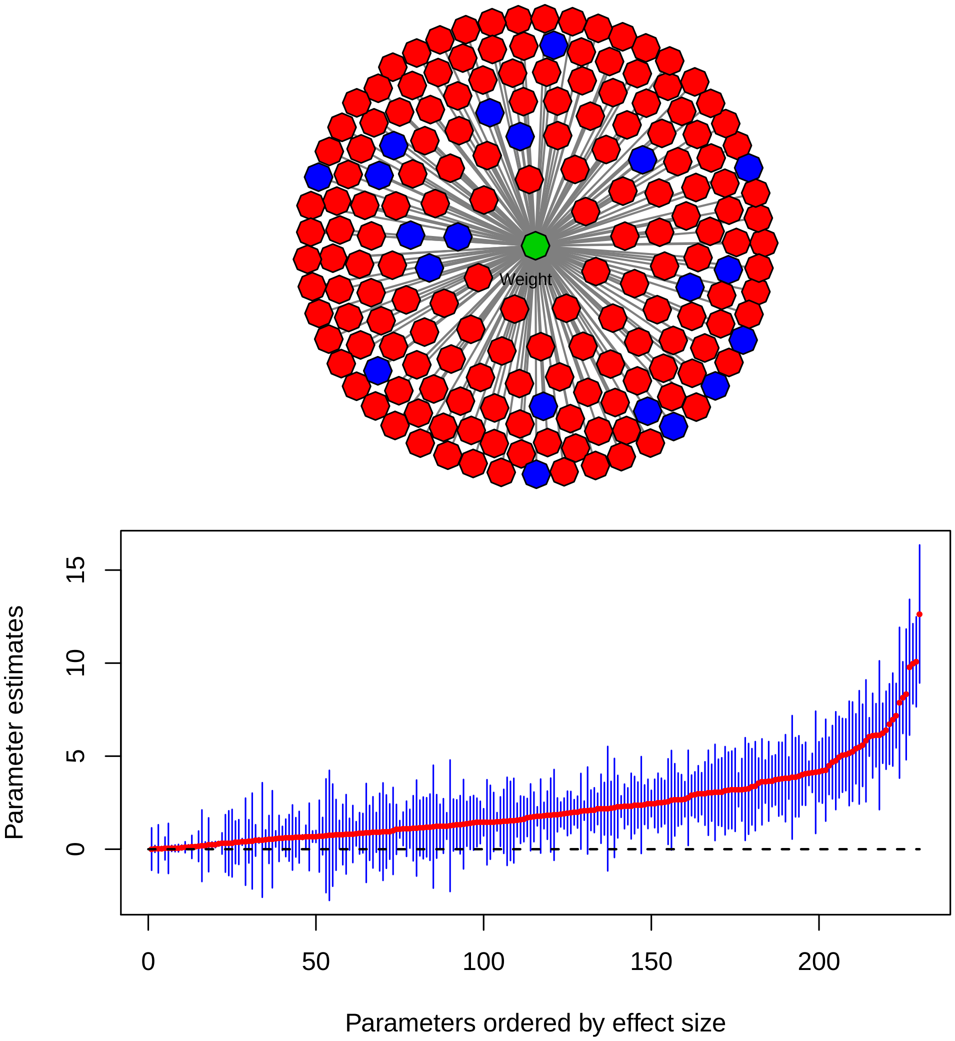

Figure S2 - Genetic marker and gene expression traits identified by the adaptive lasso with 10-fold cross-validation as being associated with weight in a mouse F_2 cross. Top panel: Graphical depiction of relationship between the weight of the mouse are depicted in green, and the identified set of gene expression in red, and genetic marker interactions in blue. Bottom panel: Estimates in red and 0.95 confidence intervals (vertical blue lines) of the parameters describing the strength of regression relationship between each of the gene expression or genotype features with weight in an independently fit, unpenalized multiple regression model.

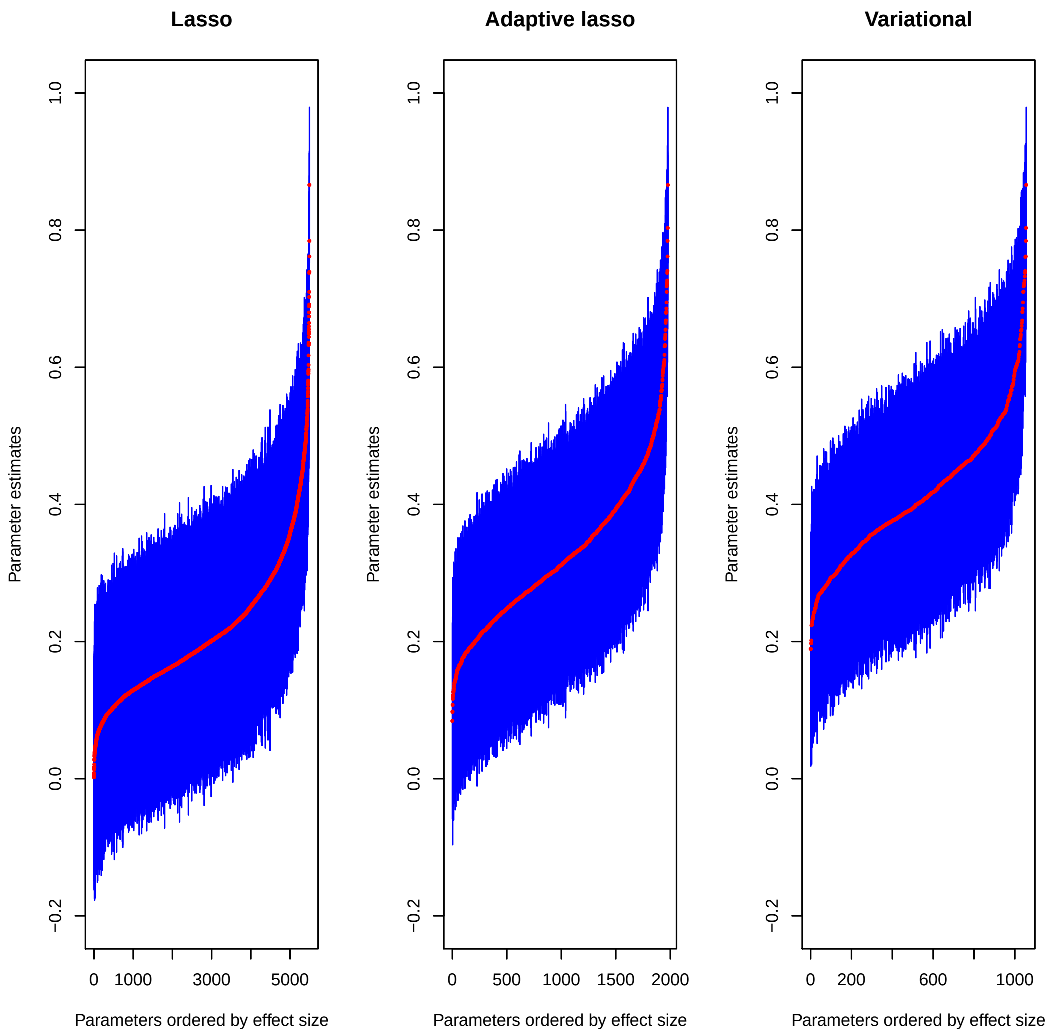

Figure S3 - Ordered estimates and confidence intervals from independently fit linear models for all interactions identified by the lasso, adaptive lasso, and variational method for simulated data of a 100 node gene expression network with a sample size of 100.

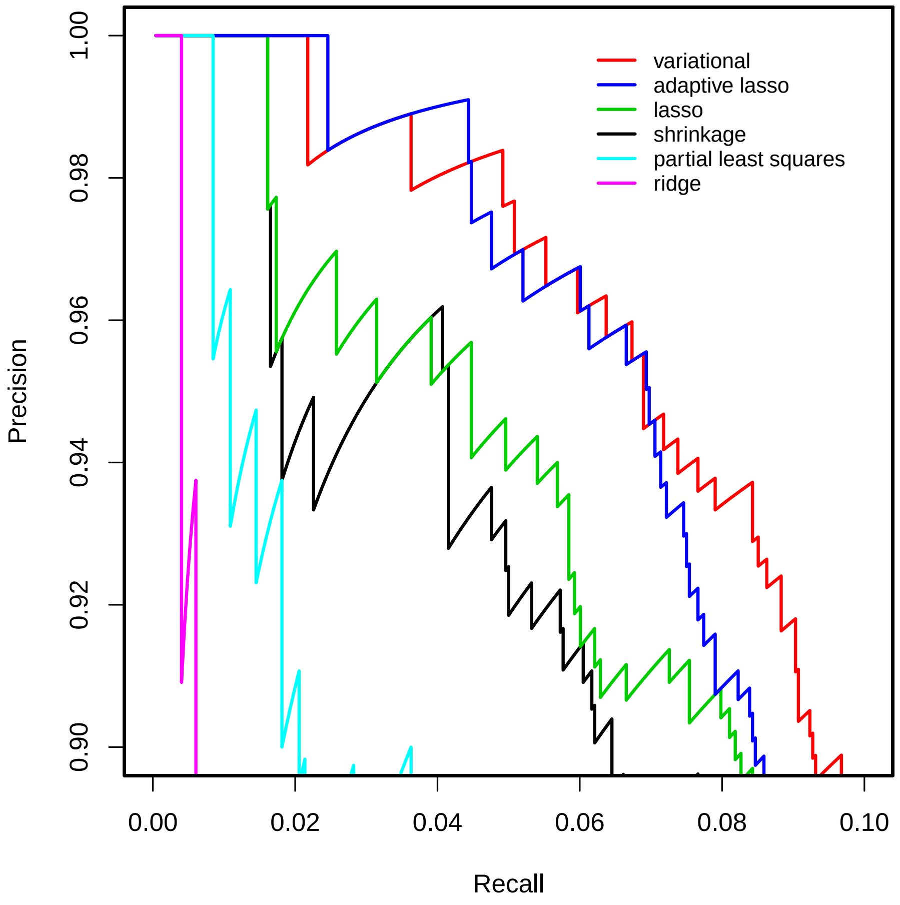

Figure S4 - A comparison across network reconstruction methods for a sparse (density of 0.05), undirected graph with 100 gene expression products, for simulated data. The precision (true discovery rate) v.s. the recall (power) is plotted for a sample size of 50 as determined by the estimated regression coefficients for each method.

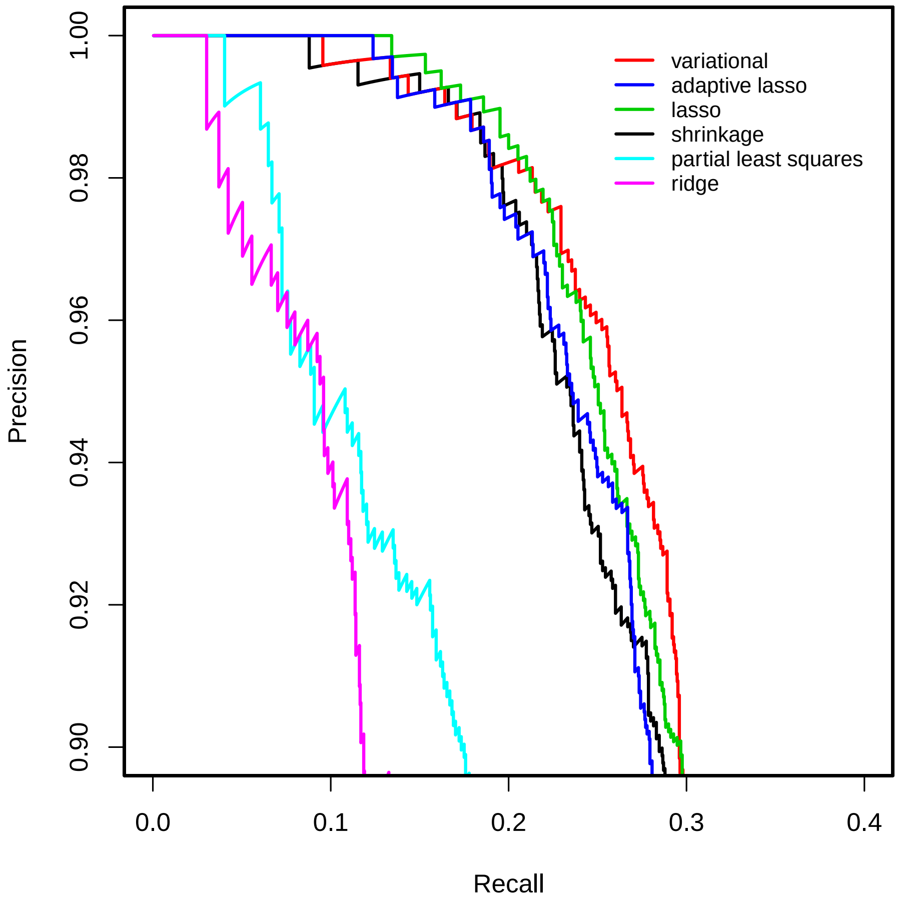

Figure S5 - A comparison across network reconstruction methods for a sparse (density of 0.05), undirected graph with 100 gene expression products, for simulated data. The precision (true discovery rate) v.s. the recall (power) is plotted for a sample size of 100 as determined by the estimated regression coefficients for each method.

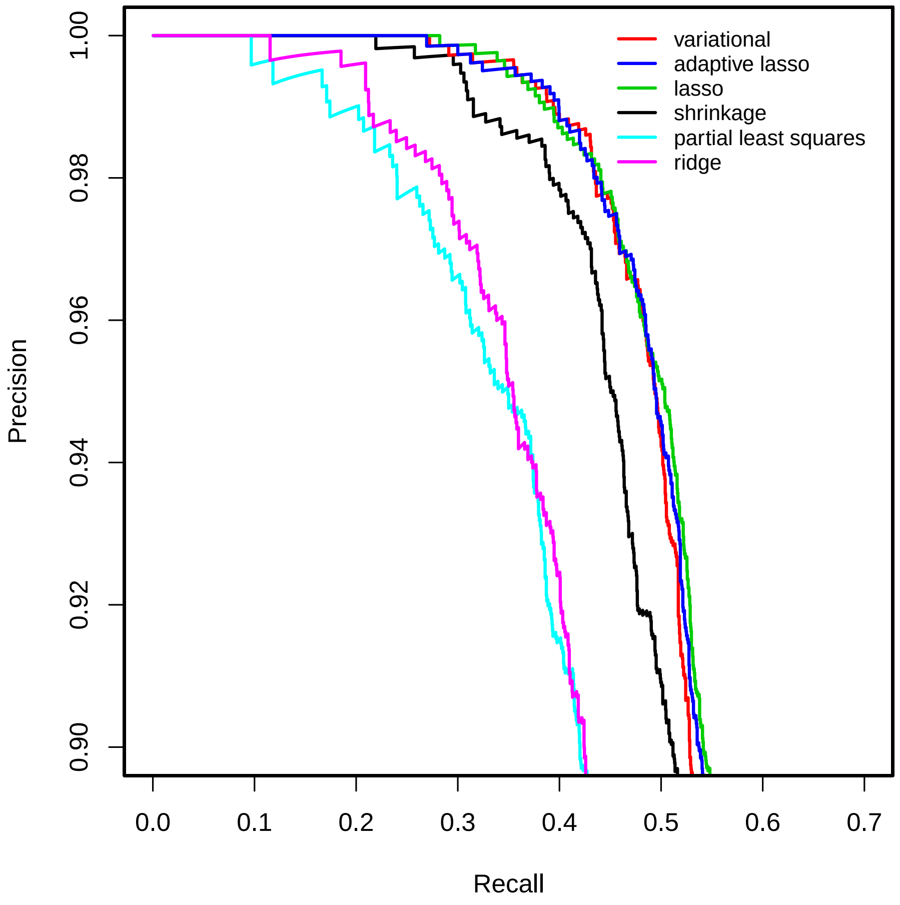

Figure S6 - A comparison across network reconstruction methods for a sparse (density of 0.05), undirected graph with 100 gene expression products, for simulated data. The precision (true discovery rate) v.s. the recall (power) is plotted for a sample size of 200 as determined by the estimated regression coefficients for each method.

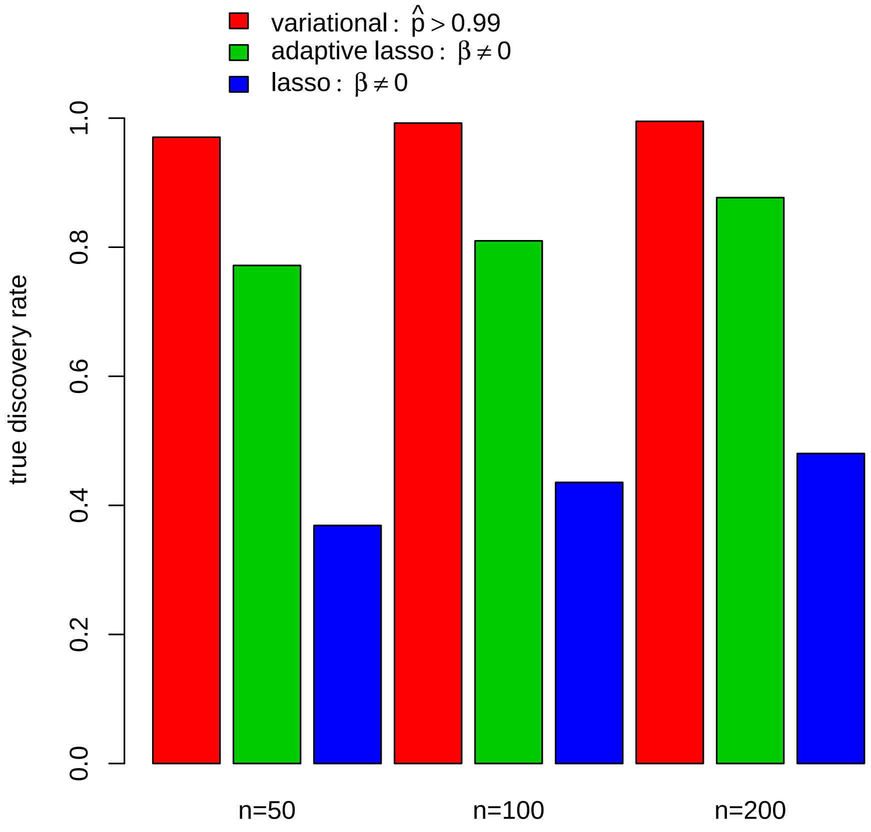

Figure S7 - True discovery rate as a function of sample size for models recovered by the variational spike and slab method, the adaptive lasso, and the lasso, under the same simulations as shown in Figures S4-S6.
